# Absence of Tumor Necrosis Factor Supports Alternative Activation of Macrophages in the Liver after Infection with *Leishmania major*

**DOI:** 10.3389/fimmu.2018.00001

**Published:** 2018-01-19

**Authors:** Shanshan Hu, Cameron Marshall, Jocelyn Darby, Wei Wei, Alan Bruce Lyons, Heinrich Körner

**Affiliations:** ^1^Menzies Institute for Medical Research, University of Tasmania, Hobart, TAS, Australia; ^2^Institute of Clinical Pharmacology, Anhui Medical University, Key Laboratory of Anti-Inflammatory and Immunopharmacology, Ministry of Education, Engineering Technology Research Centre of Anti-Inflammatory and Immunodrugs in Anhui Province, Hefei, China; ^3^School of Medicine, University of Tasmania, Hobart, TAS, Australia

**Keywords:** *Leishmania major*, liver, tumor necrosis factor, monocytes, IL-6

## Abstract

The absence of tumor necrosis factor (TNF) causes lethal infection by *Leishmania major* in normally resistant C57BL/6J (B6.WT) mice. The underlying pathogenic mechanism of this fatal disease has so far remained elusive. We found that B6.WT mice deficient for the *tnf* gene (B6.TNF^−/−^) displayed not only a non-healing cutaneous lesion but also a serious infection of the liver upon *L. major* inoculation. Infected B6.TNF^−/−^ mice developed an enlarged liver that showed increased inflammation. Furthermore, we detected an accumulating monocyte-derived macrophage population (CD45^+^F4/80^+^CD11b^hi^Ly6C^low^) that displayed a M2 macrophage phenotype with high expression of CD206, arginase-1, and IL-6, supporting the notion that IL-6 could be involved in M2 differentiation. In *in vitro* experiments, we demonstrated that IL-6 upregulated M-CSF receptor expression and skewed monocyte differentiation from dendritic cells to macrophages. This was countered by the addition of TNF. Furthermore, TNF interfered with the activation of IL-6-induced gp130-signal transducer and activator of transcription (STAT) 3 and IL-4-STAT6 signaling, thereby abrogating IL-6-facilitated M2 macrophage polarization. Therefore, our results support the notion of a general role of TNF in the inflammatory activation of macrophages and define a new role of IL-6 signaling in macrophage polarization downstream of TNF.

## Introduction

The infectious disease leishmaniasis has been identified as a “neglected tropical disease” by the WHO and affects a significant number of people worldwide. The infection is caused by members of the intracellular protozoan parasite genus *Leishmania* spp. (1) and occurs when parasites are transmitted to their human hosts by *Phlebotomus* spp. or *Lutzomyia* spp. sandfly vectors during their blood-meal (2). Depending on the specific parasite species and the immune response of the host, infection with *Leishmania* spp. results in a variety of clinical manifestations that can range from self-healing skin lesions to progressive and ultimately fatal infections of visceral organ such as the spleen and liver (3). In the mammalian host, the parasites reside in their amastigote form within macrophages and dendritic cells (DCs) (4). The experimental model of cutaneous leishmaniasis, which is based on a subcutaneous inoculation with isolates of the species *Leishmania major* features a strong genetic dichotomy. Mice of a C57BL/6 background (B6.WT) display a localized, self-healing infection characterized by interferon-γ (IFN-γ) production, while mice of the BALB/c background respond to infection with a preferential production of Th2 cytokines, such as IL-4, IL-10, and IL-13. These mice succumb to the infection after a progressive course of disease and a marked visceralization of the pathogen. This genetic dichotomy as a basis of resistance or susceptibility was instrumental in the development of the T helper (Th)1/Th2 paradigm (1).

The early expression of these respective cytokines has a profound effect on the activation state of macrophages, the predominant host cells of these pathogens. The T cell and NK cell cytokine IFN-γ triggers a classical activation that equips macrophages located in skin and local draining lymph node with mechanisms such as an upregulation of inducible nitric oxide synthase (iNOS) that allow an effective response to the challenge (5). In contrast, the cytokines IL-4, IL-10, and IL-13 result in an alternative activation in macrophages (6) that allows them to contribute to tissue remodeling and wound healing (7). While these cytokines have clearly accepted general roles in the establishment of an immune response to pathogens the proinflammatory cytokine tumor necrosis factor (TNF) has been seen as a cofactor that had an important but limited role in the immune defense (8). Nevertheless, infection experiments using *L. major*, specifically the BNI substrain, and other intracellular pathogens demonstrated a strong protective effect of TNF since *L. major* BNI infected TNF-deficient mice succumbed rapidly to the parasites despite a strong Th1-type response (9, 10). Recent observations have allowed an insight in the underlying deficiencies that cause this susceptibility. It could be demonstrated that in the *L. major* model TNF is necessary to prevent an ill-timed accumulation of alternatively activated macrophages concurrently to classically activated macrophages thus indicating a new and unique role for TNF (11). In the absence of TNF an elevated accessibility of arginase-1 (Arg-1) promoter and enhancer structures permitted a hyper-expression of Arg-1 and caused a subsequent lack of nitric oxide (NO) production presumably due to competition between the two enzymes iNOS and Arg-1, that depleted their common substrate l-arginine (12).

This mechanistic model of TNF-dependent restriction of alternative activation and the consequences for a host that lacks TNF has been established in skin and draining lymph nodes of *L. major* BNI infected mice (12). Other organs such as spleen and liver also show significant reproduction of parasites which is not detectable in TNF-positive hosts. The immune response in these organs has not yet been investigated in more detail and we hypothesized that we would also find increased alternative activation of macrophages. By analyzing *L. major* BNI infection in the liver, we found a comparatively low iNOS expression in B6.TNF^−/−^ macrophages and an accumulation of a myeloid population that exhibited an alternatively activated-like macrophage phenotype, with high expression of Arg-1, CD206 and IL-6. In an *in vitro* assay, we demonstrated that bone marrow-derived DCs treated with IL-6 increased the expression of M-CSFR and generated less CD11c^+^ cells, while adding TNF reinstated CD11c^+^ cell generation and concurrently inhibited M-CSFR expression. Furthermore, we could show that both TNF and IL-6 had a regulatory effect on M2 macrophage differentiation which depends on modulation of mIL-6/gp130/signal transducer and activator of transcription (STAT) 3 or IL-4-STAT6. These findings of our study are emphasizing again that expression of TNF is critical to preventing a spread of parasites to visceral organs.

## Results

### Progressive Liver Infection by *L. major* BNI in B6.TNF^−/−^ Mice

**Figure 1:**
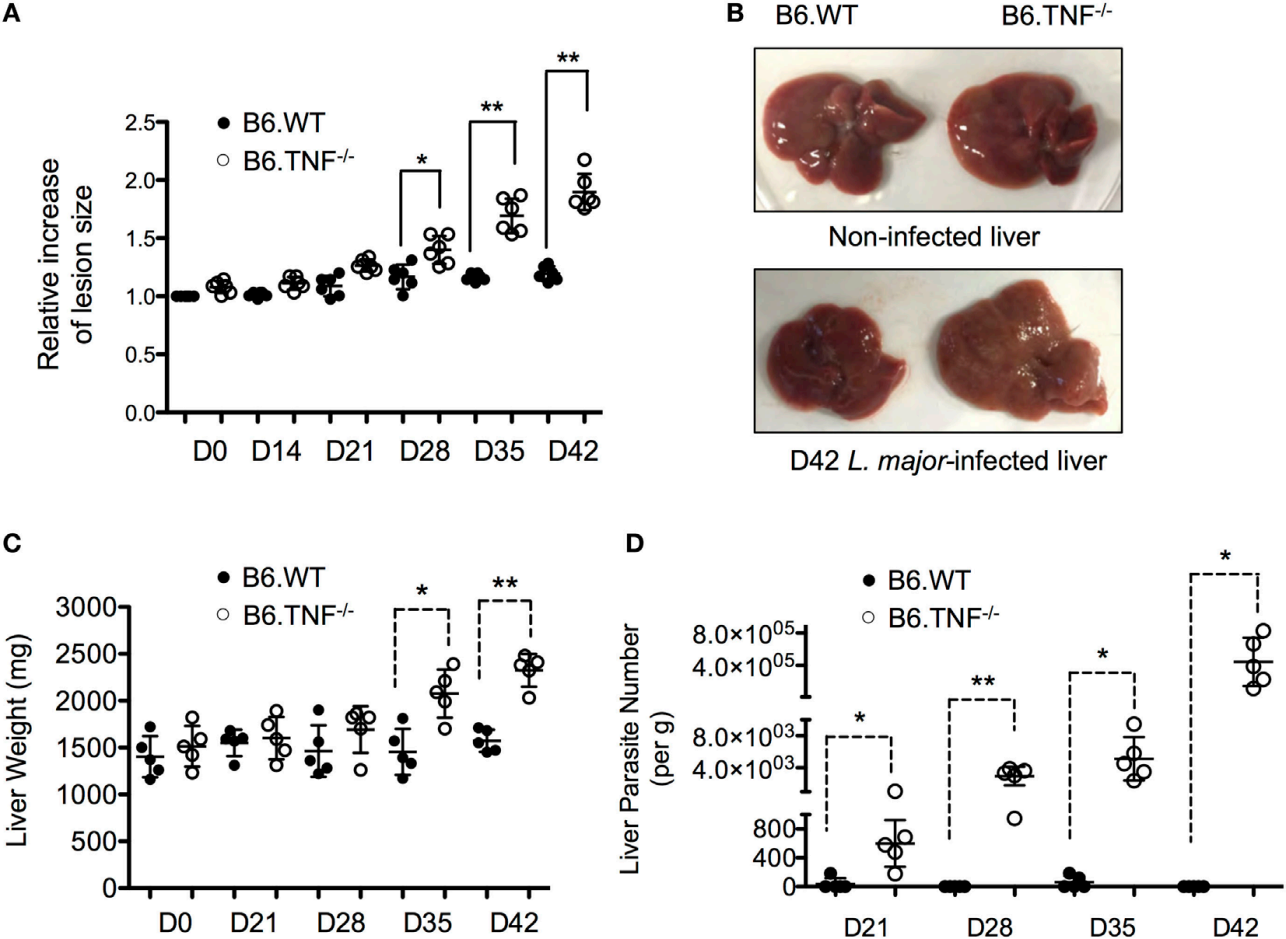
Liver enlargement and increased parasite burden in B6.TNF^−/−^ mice. While the lesion and liver size and weight remained initially identical between B6.WT and B6.TNF^−/−^ genotypes it increases significantly in footpads **(A)** and liver **(B,C)** of B6.TNF^−/−^ mice 42 days after *L. major* BNI infection. Six B6.WT and B6.TNF^−/−^ mice were used to determine lesion size **(A)**, and five mice were used to determine liver weight at each time point **(C)**. Error bars represent the mean ± SD from one representative of three independent experiments. The *p*-values were calculated using a two tailed Mann–Whitney *U*-test (*p < 0.05, **p < 0.01). **(C)** The number of viable parasites in the liver tissue of B6.WT and B6.TNF^−/−^ mice was determined by limiting dilution analysis **(D)**. The mean parasitic burden in the liver tissue of five mice from one representative experiment is shown. One circle represents one animal (black: B6.WT; white: B6.TNF^−/−^). The results were confirmed by the other two independent replications. All data are represented as mean ± SD. Significance was calculated using a two tailed Mann–Whitney *U*-test (*p < 0.05, **p < 0.01).

Infection of C57BL/6 mice that lack an expression of TNF with *L. major* BNI results in a progressive course of disease and visceralization while B6.WT mice contain the infection and recover spontaneously (10). In our infection experiments, a significant lesion was observed in both B6.WT and B6.TNF^−/−^ mice from day 21 after infection. While the footpads swelling remained moderate in B6.WT mice and subsided after day 35, B6.TNF^−/−^ mice showed a progressively increasing footpad swelling (Figure 1A). Additionally, in B6.TNF^−/−^ mice the infection spread to visceral organs such as the liver resulting in a mild hepatomegaly (Figure 1B) and a significant increase in liver weight (Figure 1C). Parasites in the liver were detected from day 21 after infection increasing in number to more than 3 × 10^5^ parasites per gram liver tissue at day 42 postinfection (p.i.), while the liver of infected B6.WT mice remained essentially parasite free (Figure 1D).

### Discrete Inflammatory Foci in the Liver of B6.TNF^−/−^ Mice during Cutaneous Leishmaniasis

**Figure 2:**
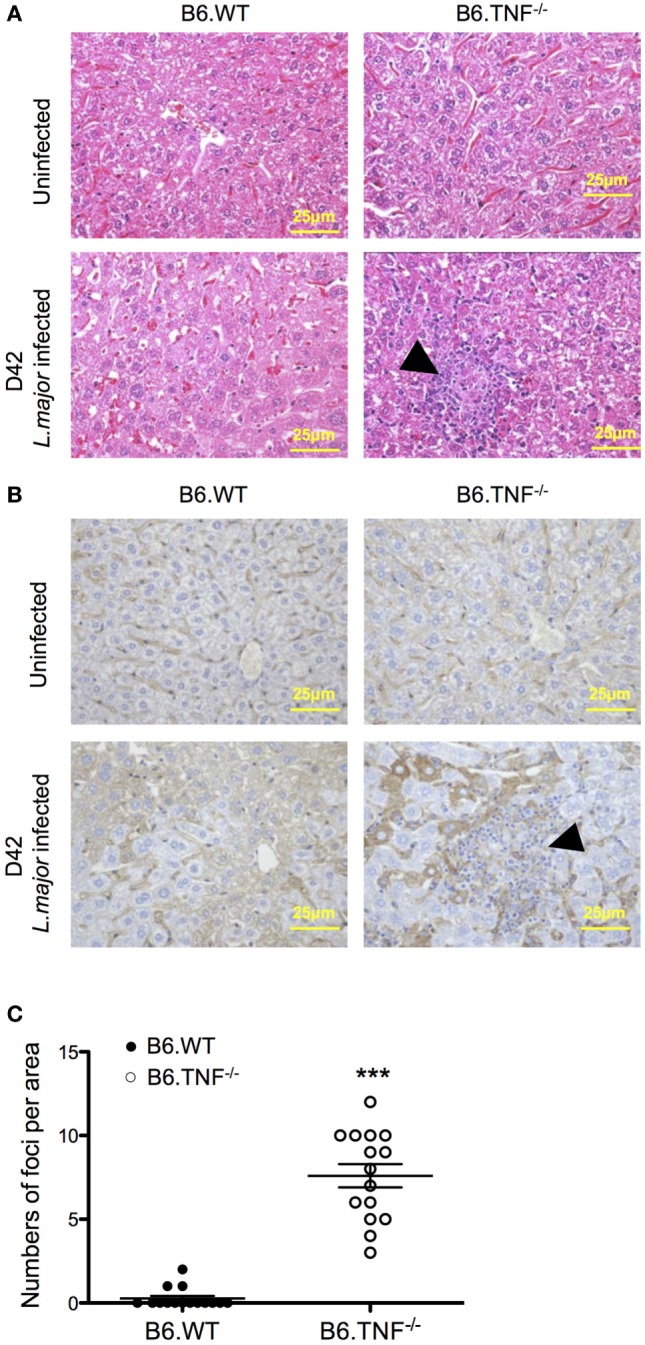
Liver morphology of B6.WT and B6.TNF^−/−^ mice. **(A)** Representative H&E-stained liver sections of 15 B6.WT and B6.TNF^−/−^ mice are shown before infection and at day 42 after infection. B6.TNF^−/−^ mice show an increased infiltration of inflammatory cells and the presence of inflammatory foci (arrowhead; magnification 400×). **(B)** Liver tissue from B6.WT and B6.TNF^−/−^ mice (uninfected and 42 days after infection). At day 42 after infection, CD68 immunostaining is markedly increased in B6.TNF^−/−^ mice compared to the B6.WT group. Bar = 25 μm for all the pictures. **(C)** The number of inflammatory foci per area is shown. The *p*-values were calculated using two tailed Mann–Whitney *U*-test (***p < 0.001).

In both B6.WT and B6.TNF^−/−^ mice normal, uninfected liver tissue consists of hexagonal hepatocytes radiating from the region of the central vein toward the periphery. From day 35 after infection, a point in time coinciding with a significant increase of liver weight and parasitic burden (Figures 1B,C), abnormal liver structures such as swelling of hepatocytes and diffusely infiltrating inflammatory cells could be detected in *L. major* BNI-infected B6.TNF^−/−^ mice (Figure 2A). Additionally, inflammatory foci appeared almost exclusively in infected gene-deficient mice (Figure 2A). The generic macrophage marker CD68 was used to determine the phenotype of the cell focus (Figure 2B). Weak hepatic expression of CD68 was observed in B6.WT and B6.TNF^−/−^ control groups, which was upregulated at day 42 after infection compared to its B6.WT counterpart. Cells that were CD68^+^ were mainly detected in areas around the borders of inflammatory foci. Taken together, these results suggested that infiltration of inflammatory cells and hepatic inflammation were significantly elevated in B6.TNF^−/−^ mice, compared to the corresponding B6.WT mice. Interestingly, the number of inflammatory foci was significantly higher in B6.TNF^−/−^ mice as compared to that in B6.WT mice (Figure 2C).

### Cytokines Levels in Serum of B6.WT and B6.TNF^−/−^ Mice after Infection

Cytokine levels in the serum are an indication of the type of immune response to *L. major* BNI (9). The titers of monocyte chemoattractant protein (MCP)-1, IL-6, IFN-γ, IL-10, TNF, and IL-12 (p70) were determined in the serum of *L. major* BNI-infected B6.WT and B6.TNF^−/−^ mice using a cytokine bead array (CBA) and compared to uninfected controls.

**Figure 3:**
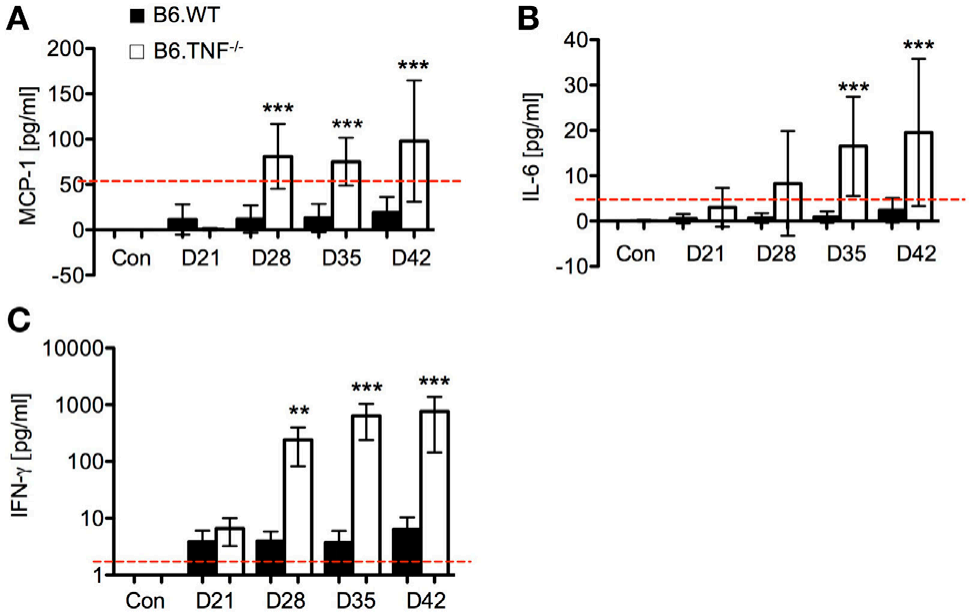
Cytokine secretion as measured by cytokine bead array using flow cytometry. Serum levels of **(A)** monocyte chemoattractant protein-1 (MCP-1), **(B)** IL-6, and **(C)** interferon-γ (IFN-γ) were measured over the course of *Leishmania major* infection between B6.WT and B6.TNF^−/−^ mice. The red dashed lines represent the limit of detection for each cytokine (IL-6 5 pg/ml, MCP-1 52.7 pg/ml, and IFN-γ 2.5 pg/ml). Concentrations were expressed and compared with the basal level found in B6.WT and B6.TNF^−/−^ mice without infection. Each value represents the mean of three independent experiments and each experiment was performed in five mice. Error bars denote SD. The *p*-values were calculated using two tailed Mann–Whitney *U*-test (**p < 0.01, ***p < 0.001).

Serum levels of the proinflammatory cytokines MCP-1, IL-6, and IFN-γ increased significantly in B6.TNF^−/−^ mice over the course of disease (Figure 3). High levels of MCP-1 were detected in the B6.TNF^−/−^ mice after day 28 after infection (Figure 3A) indicating that *L. major* BNI-induced inflammation increased the potential to recruit monocytes. IL-6 was found to be increased by 17 times at the same point in the course of infection and 9 times at day 35 and day 42 after infection when compared to infected B6.WT mice (Figure 3B). Finally, a high concentration of IFN-γ is characteristic for a Th1-type immune response in both genotypes and was determined to validate our data. Similar to previously published results (9), B6.TNF^−/−^ mice showed a significant increase of IFN-γ (Figure 3C) compared to that in B6.WT (day 28 after infection: mean ± SD, 269.45 ± 163.56 versus mean ± SD, 3.50 ± 1.49; day 35 after infection: mean ± SD, 668.39 ± 424.14 versus mean ± SD, 3.50 ± 2.46; day 42 after infection: mean ± SD, 766.03 ± 622.3 versus mean ± SD, 6.14 ± 4.11). Serum concentration of IL-10 (limit of detection 17.5 pg/ml) and IL-12p70 (limit of detection 10.7 pg/ml) remained below the detection limit for the assay in more than half of the serum samples throughout the course of infection (data not shown). TNF was only observed in B6.WT mice (data not shown).

### A Monocyte-Derived Macrophage (Mo-M) Population Accumulates in the Liver of *L. major* BNI-Infected B6.TNF^−/−^ Mice

A strong accumulation of inflammatory myeloid cells has been described in skin and draining lymph node in *L. major* BNI infection (11). Furthermore, it has been demonstrated that TNF is necessary for classically activated macrophage phenotype (12). Therefore, we analyzed the quantity and composition of infiltrating inflammatory cells in the liver of B6.WT and B6.TNF^−/−^ mice using comprehensive flow cytometric analysis with two different panels over the course of infection.

Three distinct subsets were characterized based on the expression of CD11b, Ly6C, CD45, and F4/80 as described previously (13). The liver-resident Kupffer cells (KCs) were defined as CD45^+^F4/80^+^CD11b^−^Ly6C^−^ population. Recruited inflammatory monocytes (Mo) which differentiate to Mo-M and potentially to inflammatory DCs (Mo-DC) were defined as CD45^+^F4/80^+^CD11b^low^Ly6C^hi^. Finally, Mo-M displayed the phenotype of CD45^+^F4/80^+^CD11b^hi^Ly6C^low^ (13–15) (#or gating strategy and flow cytometric identification of subpopulations see Figure S1 in Supplementary Material).

**Figure 4:**
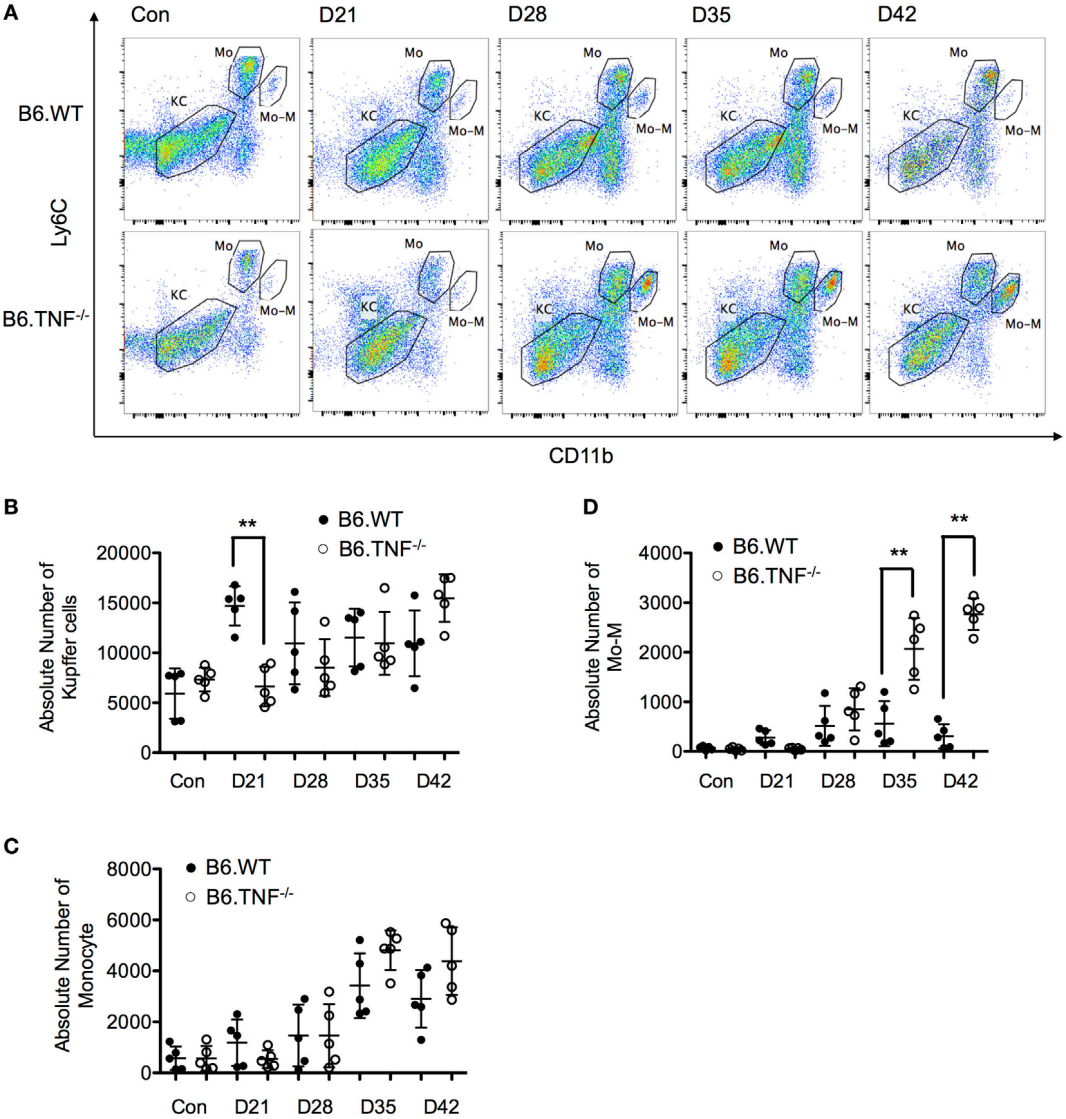
Analysis of resident and inflammatory myeloid populations in the liver in *Leishmania major* infected B6.WT and B6.TNF^−/−^ mice. **(A)** Flow cytometry analysis was used to demonstrate the presence of three different liver macrophage populations based on the markers CD45, F4/80, CD11b, and Ly6C from B6.WT and B6.TNF^−/−^ mice and to analyze the changes of these populations over the course of *L. major* BNI infection. Kupffer cells (KCs) were defined as CD45^+^F4/80^+^CD11b^−^Ly6C^−^, inflammatory monocytes (Mo) as CD45^+^F4/80^+^CD11b^low^Ly6C^hi^ and monocyte-derived macrophages (Mo-M) as CD45^+^F4/80^+^CD11b^hi^Ly6C^low^. A representative staining is shown. Quantification by flow cytometry of the total populations of **(B)** KC, **(C)** Mo, and **(D)** Mo-M from five B6.WT and B6.TNF^−/−^ mice in the course of *L. major* BNI infection is shown. Each error bar represents means ± SD from one experiment. Results were confirmed by two independent experiments. The *p*-values were calculated using two tailed Mann–Whitney *U*-test (**p < 0.01).

The number of KCs was not significantly different between the genotypes over the course of a *L. major* BNI infection except for day 21 after infection. At this point in time, the CD45^+^F4/80^hi^CD11b^−^Ly6C^−^ population was around twofold higher in B6.WT mice as compared to B6.TNF^−/−^ mice (Figures 4A,B). The number of inflammatory monocytes increased in correlation with the footpad swelling irrespective of the genotype. At day 42, the infiltrating monocytes started to decrease (B6.WT) or reached a plateau (B6.TNF^−/−^) (Figures 4A,C). At the same time, Mo-M which represented a small population in B6.WT mice were elevated significantly in B6.TNF^−/−^ mice (Figures 4A,D). To analyze the specific role of TNF in the accumulation of Mo-M, we employed TNF-competent mice of the BALB/c strain which are highly susceptible to *L. major* BNI infection and display progressive visceralization (Figures S2A,B in Supplementary Material). This control experiment showed a Mo-M population comparable to B6.WT mice and indicated that the accumulation of the Mo-M population depends on TNF.

Infection leads to a strong increase of CD11c^+^iNOS^+^TNF^+^ inflammatory DCs (here termed Mo-DC) at the infection site. These cells are also derived from inflammatory monocytes and act as effector and antigen-presenting cells. Therefore, we investigated whether the absence of TNF affects the differentiation process of Mo-DCs during *L. major*-BNI induced liver infection and we examined the expression of CD11c based on CD45^+^CD11b^hi^Ly6C^hi^ population. Mice of both genotypes had a very distinct CD11c^+^ CD11b^hi^ Ly6C^hi^ population of comparable size. In B6.TNF^−/−^ mice the populations displayed a comparatively lower expression of CD11b, Ly6C, and CD11c than B6.WT mice (Figures S3A,B in Supplementary Material), which could indicate differences of inflammatory status or maturity of Mo-DCs.

### The Mo-M Population in TNF^−/−^ Mice Display an Alternatively Activated Phenotype with High IL-6 Expression

**Figure 5:**
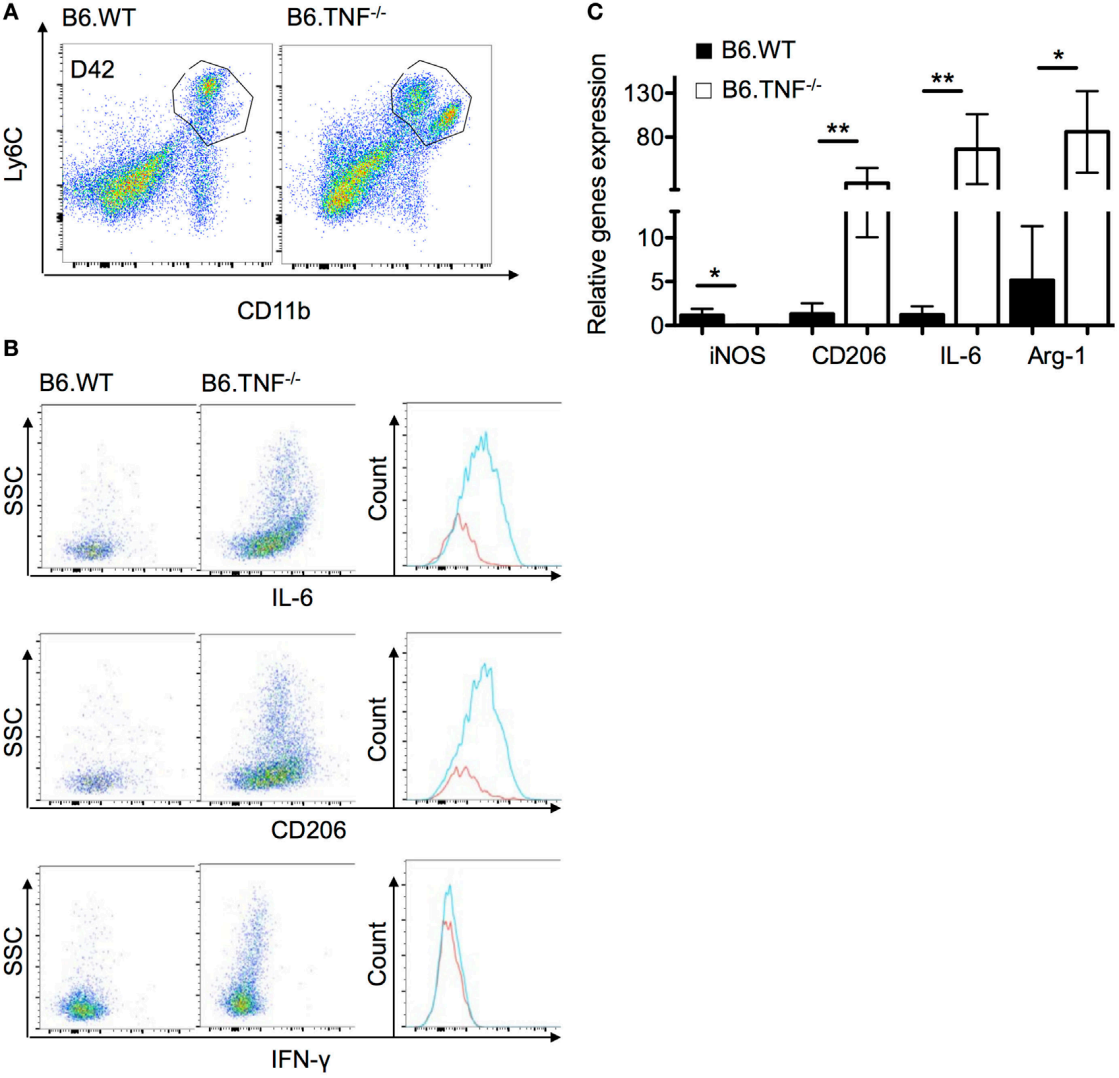
Phenotypic characterization of monocyte-derived macrophages in the liver of B6.TNF^−/−^ mice. **(A)** Gating strategy used in these experiments. **(B)** The expression of IL-6, CD206, and interferon-γ were investigated in the combined population from B6.WT and B6.TNF^−/−^ mice at day 42 p.i. using flow cytometry. **(C)** Gene expression of IL-6, CD206, inducible nitric oxide synthase, and arginase-1 relative to β-actin expression in the combined population of B6.WT and B6.TNF^−/−^ mice at d42 p.i. Each error bar represents the means ± SD from five mice in one experiment, and results were confirmed by further two independent experiments. The *p*-values were calculated using a two tailed Mann–Whitney *U*-test (*p < 0.05, **p < 0.01).

As shown above, B6.TNF^−/−^ mice fail to clear the parasites from the liver and display an accumulation of a TNF-specific, unique Mo-M accumulation. To further investigate this Mo-M population in B6.TNF^−/−^ mice during murine leishmaniasis, we followed the previously used gating strategy and combined both CD11b^+^Ly6C^+^ populations since CD11b^high^Ly6C^low^ are missing in B6.WT mice (Figure 5A). We characterized the phenotypes using intracellular flow cytometry of the markers IL-6, CD206 and, as control for the quality of the sort, IFN-γ. The intracellular antigens were depicted against SSC (Figure 5B). Corresponding to the increased IL-6 secretion in serum of infected B6.TNF^−/−^ mice, an increased level of IL-6 expression was detected in liver macrophages. Although IL-6 is regarded as proinflammatory cytokine, it could be involved in the establishment of a Th2 response which in turn, could modulate the activation pathway of the macrophage differentiation. Additionally, the macrophage mannose receptor CD206, was strongly upregulated. As expected, there was no difference in the presence of IFN-γ between B6.WT and B6.TNF^−/−^ because IFN-γ is not produced by Mo or Mo-M. In summary, we found SSC, IL-6 and CD206 increased in the combined Mo and Mo-M of B6.TNF^−/−^mice, indicating that this population comprises are large proportion of alternatively activated macrophages in B6.TNF^−/−^ mice during *L. major* BNI infection. The detection of SSC^high^ cells in B6.TNF^−/−^ mice was striking and indicates a marked presence of cells with high granularity that could represent infected cells.

Previously, it had been shown that a CD11b^hi^ Ly6C^low^ myeloid population harbored a markedly increased number of parasites in skin and draining lymph nodes (11). Because infected cells with a large burden of parasites are fragile and difficult to detect using flow cytometry, we sorted the distinct Mo and Mo-M populations from B6.WT and B6.TNF^−/−^ mice and conducted a Romanowsky stain (Diff-Quik). There was no distinctive visible difference between Mo and Mo-M cells. In B6.WT mice there were no visible parasite associated with macrophages. In contrast, in both CD11b^+^Ly6C^hi^ and CD11b^+^Ly6C^low^ macrophage populations isolated from B6.TNF^−/−^ mice parasites could be detected inside and on the surface of macrophages (Figure S4 in Supplementary Material).

To further analyze the Mo-M population in the liver during *L. major* BNI infection, we isolated these cells and characterized their phenotype with regard to the gene expression of these marker molecules using qPCR. We confirmed our flow cytometry results and showed that IL-6 and the alternative activation markers including Arg-1 and CD206 were significantly higher expressed in B6.TNF^−/−^ mice as compared to B6.WT mice. In contrast to previous results, in the liver iNOS expression was decreased significantly in B6.TNF^−/−^ mice (Figure 5C).

### Macrophage Activation Is Correlated with Parasitic Burden in Liver

**Figure 6:**
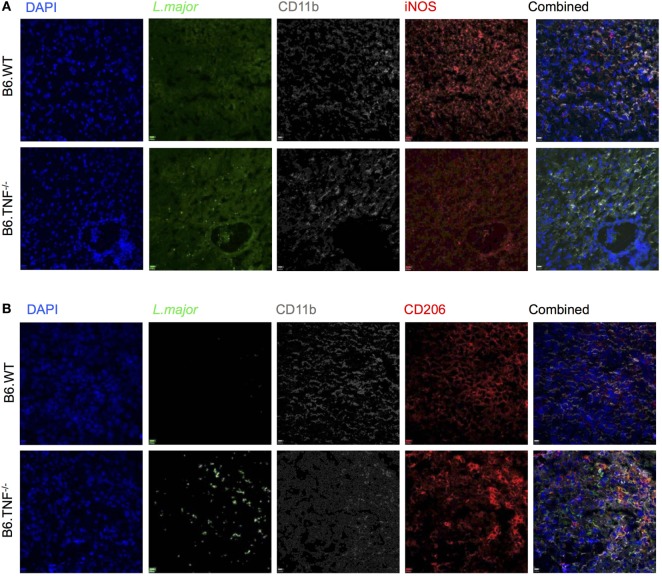
Inducible nitric oxide synthase (iNOS) and CD206 expression in B6.TNF^−/−^ mice. Immunofluorescence staining of **(A)** CD11b, iNOS, and *Leishmania major* and **(B)** CD11b, CD206, and *L. major* in liver tissue of B6.WT (upper panel) and B6.TNF^−/−^ mice (lower panel) 42 days after infection. Single color staining is shown for *L. major* (green), CD206 and iNOS (red), CD11b (gray), and DAPI (blue). The single colors have been merged. The figure represents one of three independent experiments.

The regulation of myeloid iNOS expression after challenge *in vivo* is complex and influenced by factors such as pathogen, genetic background and cytokine environment. Therefore, we examined the response of CD11b^+^ cells to *L. major* BNI in the livers of B6.WT and B6.TNF^−/−^ mice (Figures 6A,B). Mice from both genotypes showed a strong presence of CD11b^+^ cells at day 42 after infection while *L. major* amastigotes could only be detected directly in B6.TNF^−/−^ mice (Figures 6A,B). The absence of *L. major* in the livers of B6.WT mice was correlated with a strong iNOS expression, while in the absence of TNF, iNOS expression was decreased (Figure 6A) similar to the situation in susceptible BALB/c mice (Figure S2A in Supplementary Material) while CD206 expression was increased and associated with a strong presence of *L. major* (Figure 6B). Thus, these results suggested a presence of alternatively activated macrophages during leishmanial infection in the liver in B6.TNF^−/−^ mice.

### TNF and IL-6 Modulate the Process of Monocyte Differentiation to Macrophage or DC *In Vitro*

**Figure 7:**
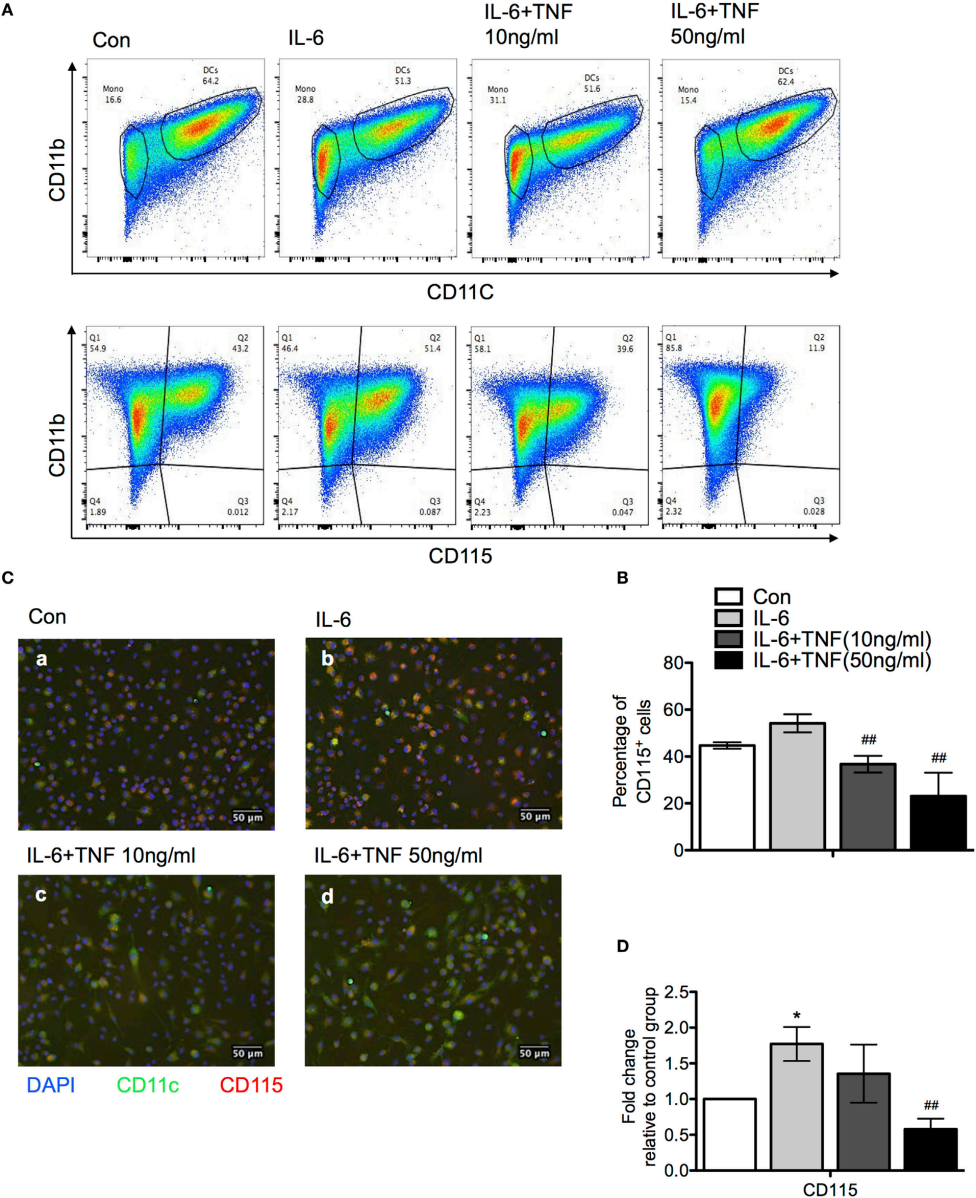
The effect of tumor necrosis factor (TNF) and IL-6 in monocyte differentiation. **(A)** Bone marrow monocytes were cultured for 4 days with granulocyte macrophage colony-stimulating factor and IL-4. IL-6 and/or TNF were added after day 4. At day 7, differentiation was monitored using the expression of CD11b, CD11c, and M-CSFR (CD115) using flow cytometry. A representative result is shown. **(B)** Quantification the results of a CD11b versus M-CSFR (CD115) staining of three independent differentiation experiments. The *p*-values were calculated using one-way ANOVA (*p < 0.05). **(C)** Immunofluorescence photographs represent sections of cells from each culture condition labeled for M-CSFR (red) CD11c (green) and DAPI (blue). **(D)** qPCR data revealed M-CSFR gene expression in cells treated with IL-6 and TNF. The results are representative of three independent experiments. Means ± SD were calibrated to median values of three experiments. The *p*-values were calculated using one-way ANOVA and Tukey’s comparison test (*p < 0.05 and ^##^*p* < 0.01).

One of the cytokines which was upregulated significantly in the liver is IL-6 (compare Figure 3). The presence of IL-6 has been shown to modulate the differentiation of monocytes and skew the developmental outcome to macrophages rather than DC, which can be reversed by TNF (16). The observed overexpression of IL-6 in B6.TNF^−/−^ mice could facilitate the increased presence of alternatively activated macrophages in the liver. To test this hypothesis, we decided to use mouse bone marrow-derived macrophages to analyze their differentiation in the context of TNF and IL-6 *in vitro*. Bone marrow cells of B6.WT mice were cultured with IL-4 and granulocyte macrophage colony-stimulating factor (GM-CSF) resulting in 64.2% CD11b^+^CD11c^+^ DC. The addition of IL-6 at day 3 of culture reduced the generation of cells with this phenotype to 51% (Figure 7). In contrast, culturing cells in the presence of a combination of IL-6 and 50 ng/ml TNF allowed the expression of CD11c to be restored to previous levels, indicating TNF reversed the differentiation process from monocytes to DCs which was inhibited by IL-6 (Figures 7A,B). The combination of GM-CSF and IL-4 potentially caused ectodomain shedding of the M-CSF receptor, inhibiting the differentiation of macrophages from monocytes (17). Thus, we next examined the level of M-CSFR expression when treated with IL-6 and TNF using flow cytometry, immunofluorescence and qPCR (Figures 7B,C). Adding IL-6 resulted in an increased level of M-CSFR expression compared to the control group which was consistent with the increased accumulation of DCs (Figure 7A). After an additional exposure to TNF, IL-6-treated DCs showed a markedly reduced M-CSFR level (Figure 7A). Using immunofluorescence DCs showed increased expression of M-CSFR when treated with IL-6 alone, but its CD11c expression was reduced compared to the control group (Figure 7B). An exposure of cells to IL-6 and TNF-α together reduced the expression of M-CSFR and CD11c was elevated. These results were confirmed using qPCR (Figure 7C).

### A Regulatory Balance of TNF and IL-6 Mediate Alternative Macrophage Polarization

**Figure 8:**
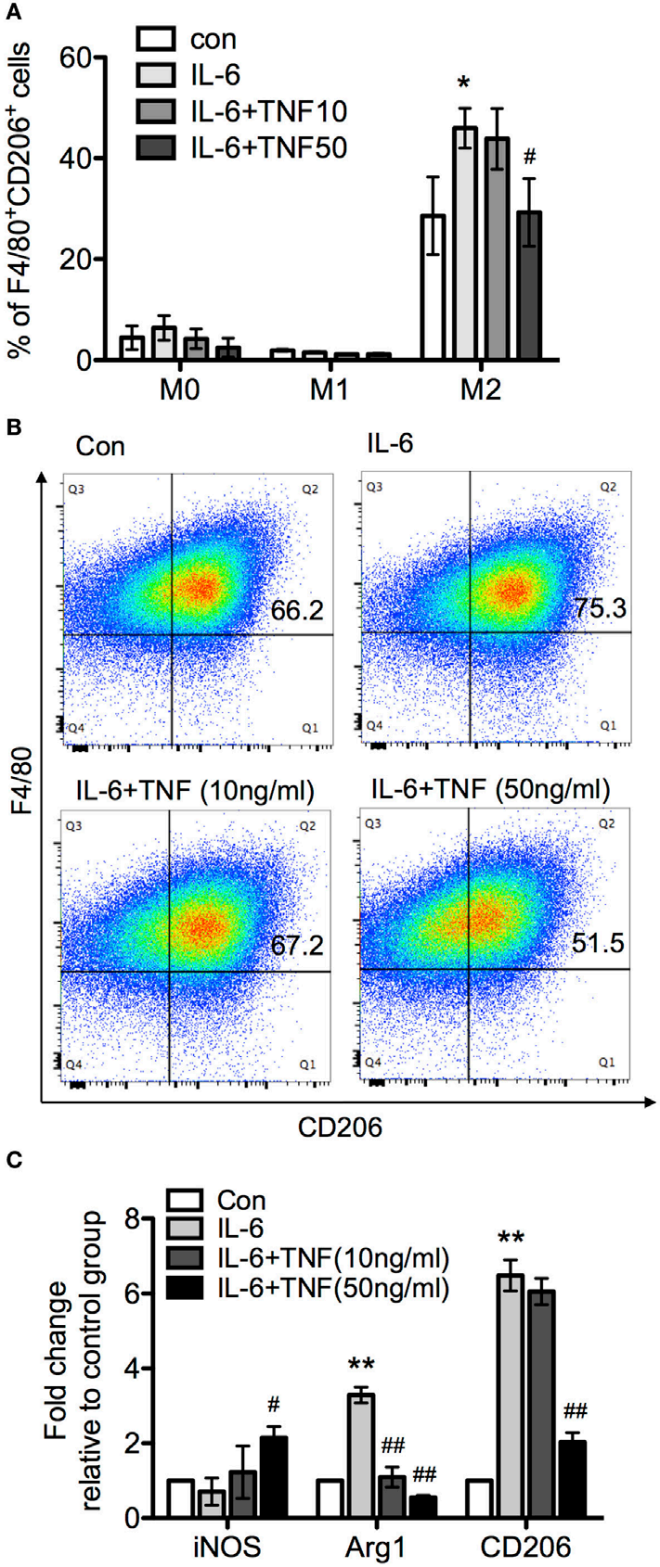
The effect of tumor necrosis factor (TNF) and IL-6 in macrophage differentiation. Bone marrow-derived macrophages were cultivated in the presence of macrophage colony-stimulating factor and harvested after 8 days. Subsequently, macrophages were exposed to LPS and interferon-γ or IL-4 for 24 h to generate M1 and M2 macrophages. The upregulation of the marker molecules F/80 and CD206 was quantified. Unchanged macrophages were considered as M0 phenotype. **(A)** M0, M1, and M2 macrophages were incubated with IL-6 or IL-6/TNF, and differentiation was analyzed. All the data are presented as means ± SD after normalization to control group values of three experiments. The *p*-values were calculated using one-way ANOVA and Tukey’s comparison test (*p < 0.05 when compared to the control group, and ^#^*p* < 0.05 when comparing to the IL-6-treated group). **(B)** Flow cytometry of cells from each culture condition using the macrophage markers F4/80 and CD206. **(C)** Quantification of the expression of inducible nitric oxide synthase (iNOS), arginase-1 (Arg-1), and CD206 in M2 macrophages treated with IL-6 and TNF using qPCR. Results are representative of three independent experiments, and presented as mean ± SD normalized with regard to the control group. The *p*-values were calculated using one-way ANOVA and Tukey’s comparison test (**p < 0.01 when compared to control group, and ^#^*p* < 0.05, ^##^*p* < 0.01 when compared to the IL-6-treated group).

Since TNF skewed IL-6-driven differentiation from macrophage to DC, we next examined whether TNF and IL-6 also affected alternative activation as represented by comparing F/80 and CD206 expression patterns. Treatment of the three different types of macrophages with IL-6 alone did not change M0 and M1 phenotype, based on the expression of F4/80^+^CD206^+^ compared to the control group (Figure 8A). However, a significant increase of F4/80^+^CD206^+^ population was observed after IL-4 treatment (Figures 8A,B). An analysis using qRT-PCR also revealed increased expression of CD206 and Arg-1 mRNA, and a reduced iNOS mRNA expression (Figure 8C), which indicated IL-6 only interfered with IL-4-induced alternative activation. Furthermore, when applied as cotreatment with IL-6 and TNF, M0 and M1 were still not affected based on the parameters assessed, compared to IL-6-treated group. The size of the F4/80^+^CD206^+^ population as well as the expression of CD206 and Arg-1 mRNA were significantly reduced with increased concentration of TNF but the iNOS mRNA expression was upregulated in the presence of TNF-α and IL-6 (Figure 8C).

### Effect of a Blockade of the Interaction of TNF and IL-6

**Figure 9:**
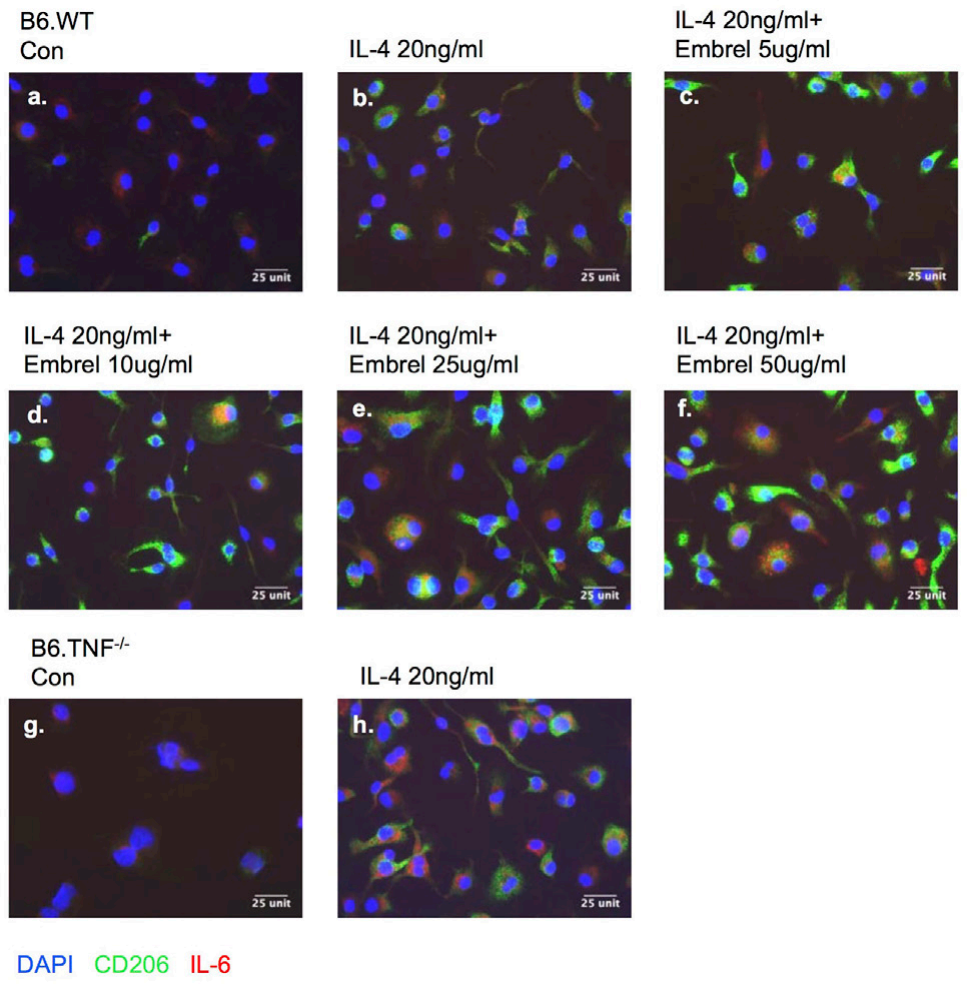
Blocking tumor necrosis factor skews macrophage differentiation. Bone marrow-derived macrophages from B6.WT **(A–F)** and B6.TNF **(G,H)** mice were cultured with IL-4 and different concentrations of etanercept (Enbrel). Immunofluorescence photographs represent cells from each culture condition labeled for IL-6 (red), CD206 (green), and DAPI (blue). One of three independent experiments is shown.

To further dissect the regulatory effect between TNF-α and IL-6 in M2 macrophage activation, we used a human TNF-receptor:Fc fusion protein (Enbrel) which is a TNF inhibitor with human/mouse cross-reactivity (18). After 8 days in M-CSF-containing medium the bone marrow derived undifferentiated macrophages expressed IL-6 (Figures 9A,G). Additional exposure to IL-4 for 48 h induced macrophages from both B6.WT and B6.TNF^−/−^ mice to display CD206 (Figures 9B,H). The level of IL-6 was largely unchanged in B6.WT macrophages but strongly upregulated in B6.TNF^−/−^ macrophages. Interestingly, macrophages pretreated with increasing concentrations of Enbrel (5, 10, 25, 50 µg/ml) and then exposed to IL-4 not only upregulated the expression of CD206 but also increased the level of IL-6 (Figures 9C–F). Together, these data showed that IL-6 was associated with IL-4-driven alternative macrophage activation. The presence of TNF inhibited this process as indicated by a downregulation of Arg-1 and CD206. In contrast, blockade of TNF was facilitating a CD206 and IL-6 expression, supporting recent data obtained in TNF^−/−^ mice (12) and suggesting a balancing effect between TNF and IL-6 in alternative macrophage activation.

### The Regulatory Effect of the Balance between TNF and IL-6 Affects gp130/STAT3 and IL-4/STAT6 Signaling

**Figure 10:**
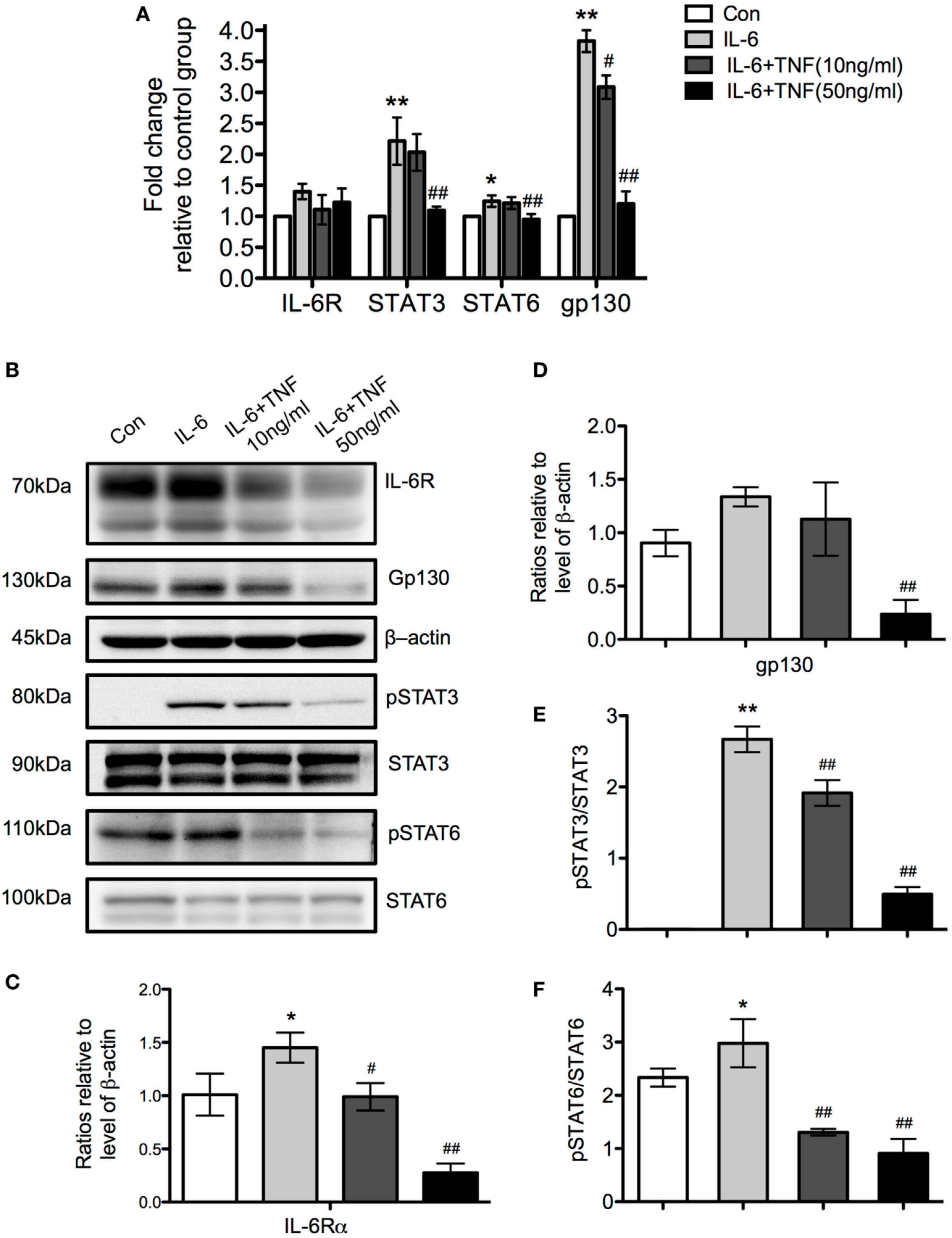
The regulatory effects of tumor necrosis factor (TNF) and IL-6 affect the expression of gp130 and IL-6R and the phosphorylation of signal transducer and activator of transcription (STAT) 3 and STAT6. **(A)** qRT-PCR analysis of different signaling molecules in M2 macrophages treated with IL-4 and TNF. Results were normalized to the control group and represent means ± SD of three experiments. Statistical analysis was performing using ANOVA with Tukey’s posttest, and the results were considered significant with a *p < 0.05, **p < 0.01 when compared to the control group, and ^#^*p* < 0.05 and ^##^*p* < 0.01 when compared to the IL-6-treated group. **(B)** Western Blot analysis of IL-6R, gp130 and b-actin as loading control. Furthermore, the transcription factors signal transducer and activator of transcription (STAT) 3 and STAT6 were analyzed as phosphorylated and non-phosphorylated proteins. One of three experiments is shown. **(C,D)** Changes of IL-6R and gp130 relative to β-actin are shown. **(E,F)** The ratio of as phosphorylated and non-phosphorylated STAT3 and 6 is depicted. Results were calibrated to the control group value and represent means ± SD of three experiments. *p < 0.05, **p < 0.01 when compared to the control group, ^#^*p* < 0.05 and ^##^*p* < 0.01 when compared to the IL-6-treated group. One-way ANOVA analysis with Tukey’s postcomparison.

Using qPCR, we examined the expression of the receptor molecules specific for IL-6, IL-6 receptor (IL-6R), and gp130, to determine which signaling pathway is active after stimulation with TNF and IL-6 in M2 macrophages. There was no significant change of IL-6R mRNA while gp130 was increased significantly in the IL-6-treated group but its expression was reduced when cells were additionally exposed to TNF (Figure 10A). A Western Blot analysis of IL-6R and gp130 showed an upregulation of these molecules in the presence of IL-6 and a significant downregulation by TNF (Figures 10B–D). Further analysis of the levels of STAT3 and 6 demonstrated that the protein expression of these transcription factors was unchanged *per se* but that the level of phosphorylation decreased in the presence of TNF (Figures 10B,E,F).

## Materials and Methods

### Mouse Strains

C57BL/6J mice (B6.WT), BALB/c mice (both Jackson Laboratories, Bar Harbor, ME, USA) and C57BL/6J mice genetically deficient for the *tnf* gene (B6.TNF^−/−^) (19) were used at 6–12 weeks of age. All mice were kept under specific pathogen-free conditions at the animal facilities of the Menzies Institute for Medical Research, Hobart, Australia. Infection was performed with sex- and age-matched B6.WT and B6.TNF^−/−^ mice. Animal care and experiments were approved by the animal ethics committee of the University of Tasmania, Hobart, Australia (Animal Ethics Number: A13934 and A13935).

### Culture of *L. major* and Infection

The virulent *L. major* strain BNI (MHOM/IL/81/FE/BNI) was kindly provided by Prof. Christian Bogdan (Institute of Microbiology, Hygiene and Immunology, Erlangen, Germany). The infectivity of the parasites was maintained by passage through the tissue of susceptible BALB/c mice as described (20). Prior to infection, the parasites were cultured *in vitro* in Novy-MacNeal-Nicolle blood agar slants in RPMI 1640 medium (Thermo Fisher Scientific, VIC, Australia) containing 10% rabbit serum (Applied Biological Products Management, SA, Australia), penicillin/streptomycin, nonessential amino acids, and 10 mM HEPES (Thermo Fisher Scientific) as described (10, 20). Mice were injected with 3 × 10^6^ stationary-phase *L. major* promastigotes bilaterally into the skin of the hind footpads. The progress of the infection was monitored by measuring the footpad swelling (lesion size) using a metric caliper (10).

Limiting dilution experiments were performed to determine the parasite burden in the infected liver. Single cell suspensions were prepared in supplemented Schneider’s media (Thermo Fisher Scientific) and serial dilutions (threefold) were pipetted across a 96-well plate with 12 replicates in an end-point titration. The plates were incubated for 10–14 days at 27°C before the number of *Leishmania*-positive wells were determined using both a light microscope Olympus CKX31 (Olympus, VIC, Australia) and a spectra Max/M2 Microplate reader (Molecular Devices, Sunnyvale, CA, USA). The parasitic burden was calculated as described (10).

### CBA Assay

Mouse whole blood was collected by cheek bleeding. Mouse serum was stored at −80°C for further use. Cytokine titers of IL-6, IL-10, MCP-1, IFN-γ, TNF, and IL-12p70 were determined in serum using the mouse inflammation CBA kit (BD Biosciences, NSW, Australia) following the manufacturer’s instructions. Samples were acquired on a BD FACSCanto II using FACSDiva 6.1 software and analyzed with FCAP Array version 1.0 software (BD Biosciences).

### Liver Mononuclear Cell Isolation

Mice were sacrificed by CO_2_ and then perfused slowly *via* the ascending aorta with 30 ml PBS and EDTA (Thermo Fisher Scientific). Livers were then removed and stored as required. Isolation of liver mononuclear cell was achieved by cutting the organ in small pieces and then digesting it in Hank’s Balanced Salt solution (Thermo Fisher Scientific) containing collagenase II (100 U/ml, Thermo Fisher Scientific) and DNase I (1 U/μl, Sigma-Aldrich, NSW, Australia) for 30 min at 37°C shaking at 200 rpm. The suspension was filtered through a 100 µm strainer (Thermo Fisher Scientific) to remove tissue debris. Cells were resuspended in PBS/BSA and mononuclear cell were isolated using a Histopaque 1083 gradient (Sigma-Aldrich). The gradient was initially centrifuged at 80 g for 3 min followed by centrifugation at 1,400 *g* for 15 min at 4°C. The cells at the interface were harvested, resuspended in 10 ml PBS and centrifuged at 600 *g* for 10 min at 4°C. The pellet was resuspended in PBS and the cell number determined in a Neubauer hemocytometer (Australian Scientific, NSW, Australia).

### Generation and Tissue Culture of Bone Marrow-Derived Macrophages and DC

BM cells were flushed from femur and tibia of uninfected B6.WT and B6.TNF^−/−^ mice and cultured in RPMI 1640 media (Thermo Fisher Scientific) supplemented as described with penicillin/streptomycin, nonessential amino acids, and 10 mM HEPES (Thermo Fisher Scientific) and either 10% of L929 tissue culture supernatant containing macrophage colony stimulating factor (M-CSF) for 7 days (21) or 10% tissue culture supernatant of GM-CSF-transfected X63-AG8 cells for 10 days (22). Recombinant mouse IFN-γ (20 ng/ml; Peprotech, Lonza, VIC, Australia) and IL-4 (10 ng/ml; Peprotech) were added into the medium for 24 h to differentiate M1 and M2, respectively. For the final 24 h, recombinant mouse IL-6 (10 ng/ml, Peprotech), TNF (Peprotech) were added. In blocking experiments increasing concentrations of a human TNFR:Fc (Enbrel; Amgen, North Ryde, NSW, USA) were added for 4 h before the addition of IL-4.

### Flow Cytometry and Cell Sorting

Multicolor flow cytometry was performed following an established protocol (23). Cells were stained first for surface marker expression with rat antimouse CD45 (Biotinylated; 30-F11; BD Biosciences), rat antimouse Ly6C (FITC; clone HK 1.4; BioLegend, WA, Australia), rat antimouse F4/80 (APC-Cy7; clone BM8; eBioscience, VIC, Australia), and rat antimouse CD11b (PerCP-Cy5.5; clone M1/70; BD Biosciences). For intracellular flow cytometry the cells were fixed with FOXP3 Fix/Perm buffer and permeabilized with FOXP3 Perm buffer (BioLegend) according to the manufacturer’s protocol. Intracellular proteins were targeted with rat antimouse CD206 (PE; clone C068C2; BioLegend), rat antimouse IL-6 (PE; clone MP5-20F3; BD Biosciences), rat antimouse IFN-γ (PE; clone XMGI-2; BD Biosciences), rabbit anti-*L. major* [clone V121 (11)], and mouse antimouse Arg-1 (PE; polyclonal antiserum; R&D Systems, Sydney, NSW, Australia). Streptavidin conjugated to V500 (BD Biosciences) was used to reveal biotinylated primary mAbs. Cells were acquired on a BD FACSCanto II flow cytometer using BD FACSDiva version 6.1.3 (BD Biosciences) and analyzed with FlowJo software version 10.1(Tree Star Inc., Ashland, OR, USA). For flow cytometric cell sorting, two populations defined by CD45^+^F4/80^+^CD11b^+^Ly6C^low^ and CD45^+^F4/80^+^CD11b^+^Ly6C^hi^ were sorted using a Beckman Coulter Astrios MoFlo. For liver DC marker comparison, CD11c (PE-Cy7; clone HL3; BD Biosciences) was used. Bone marrow-derived cells were stained with CD11b (FITC; clone M1/70; BD Biosciences), CD11c (PE-Cy7; clone HL3; BD Biosciences), F4/80 (APC-Cy7; clone BM8; eBioscience), CD206 (PE; clone C068C2; BioLegend), and M-CSFR (APC; clone AFS98; Biolegend) as experiments required.

### Immunohistochemistry

Liver tissue specimen was fixed in formalin and embedded in paraffin. Histological sections of 4 µm thickness were stained with hematoxylin and eosin using a standard protocol. The histopathological changes before and after *L. major* infection were observed using a Leica DM2500 (North Ryde, Australia). To assess the degree of inflammation, three representative inflammatory foci were imaged at 100× magnification. The number of inflammatory foci in each image was quantified and the average for each of the three representative areas per animal was then calculated.

For immunohistochemical staining, tissue sections were de-paraffinized in xylene and rehydrated. The antigens were retrieved in 10 mmol/l sodium citrate buffer (pH 9.0) for 10 min at 100°C and then cooled to room temperature before being stained. Endogenous peroxidase activity was quenched by treatment with 3% H_2_O_2_ in methanol for 10 min. The sections were blocked in protein block (X0909, Dako, VIC, Australia) for 30 min at room temperature and then incubated with a primary mAb to CD68 (ab31630, Abcam, VIC, Australia) for 1 h at 37°C. Immunoreactivity was visualized with diaminobenzidine (Dako) using the Envision system (Dako) according to the manufacturer’s protocol. The nuclei were lightly counterstained with hematoxylin solution. A negative control was prepared using the same staining procedure but was not incubated with the abovementioned primary antibodies. Images were obtained using an Olympus BX53 microscope (Olympus), and semiquantitative analysis was conducted using Image-Pro Plus software (ImageJ, USA).

Flow cytometrically sorted cells were centrifuged on slides in a Cytospin™ 4 Cytocentrifuge (Thermo Fisher Scientific) at 500 *g* for 10 min and underwent microscopical analysis after air-drying and staining with Diff-Quik reagent.

### Immunofluorescence

Liver tissue samples were dissected and rapidly frozen in Tissue-Tek optimal cutting temperature medium (VWR, QLD, Australia) in liquid nitrogen vapor and stored at −80°C. Sections (8 µm) were cut using a cryotome (Thermo Fisher Scientific), air-dried, and fixed in acetone at −20°C. Prior to staining, sections were rehydrated in PBS/1% BSA for 60 min. Sections were incubated for 60 min with a biotinylated or purified antibody, washed three times with PBS/BSA and labeled for 60 min with a secondary reagent. The primary antibodies and the second labeling reagents are described in Table 1. Sections were mounted with polyvinyl alcohol mounting media with DABCO (Sigma-Aldrich) to prevent fading. Anti-*Leishmania* antibodies (clone V121, MHOM/IL/67/Jericho II) were purified from rabbit serum (a generous gift from Emanuela Handman, WEHI, Australia) (11). Immunofluorescence images were visualized using UltraView Spinning disk confocal microscope with Velocity Software (Perkin Elmer, MA, USA). All the images were processed using ImageJ version 1.50i (ImageJ, USA).

**Table 1:** Primary and secondary antibodies used in confocal microscopy.

	Host	Target	Fluorochrome	Clone	Supplier
Primary antibodies	Rabbit	*L. major*	–	V121	M. Mack, Regensburg
	Rat	CD11b	Biotin	M1/70	BD Biosciences
	Mouse	iNOS	–	6/iNOS/NOS Type II	BD Biosciences
	Rat	CD206	–	MR5D3	BD Biosciences
	Rat	IL-6	Biotin	MP5-32C11	BioLegend

Secondary reagents	Rat	Streptavidin	Alexa Fluor 546	–	Thermo Fisher Scientific
	Donkey	Rat IgG	Alexa Fluor 647	–	Thermo Fisher Scientific
	Goat	Rabbit IgG	Alexa Fluor 488	–	Thermo Fisher Scientific
	Goat	Mouse IgG	Alexa Fluor 647	–	Thermo Fisher Scientific

### Quantitative Real-Time PCR

RNA was extracted from sorted liver macrophages and from tissue culture by using Tri-Reagent (Sigma-Aldrich) and RNA isolation mini kit (Bioline, Alexandria, Australia) according to the manufacturer’s instructions. RNA was stored in RNase-free water at −80°C. The QuantiTect Reverse Transcription Kit (Qiagen, Melbourne, VIC, Australia) was used to reverse-transcribe up to 1,000 ng total RNA. cDNA (2 µL) was amplified by Quantitative real-time PCR on the Rotor-Gene Q qPCR instrument (Qiagen) with 10 µl reactions using the SensiFAST™ SYBR No-Rox Kit (Bioline). The appropriate oligonucleotide primers were listed in Table 2, and the reaction was performed under the following conditions: samples were heated at 95°C for 3 min and amplified with 40 cycles of 95°C for 10 s, 60°C for 15 s, and 72°C for 30 s. Reactions were performed in duplicate and gene expression levels were normalized to β-actin. Relative gene expression between samples was calculated using the 2^−ΔΔCt^ calculation method.

**Table 2:** Primers used for qPCR characterizing monocytes.

Gene	Forward primer	Reverse primer	Product size (bp)
Arg-1	ATGGAAGAGACCTTCAGCTAC	GCTGTCTTCCCAAGAGTTGGG	224
iNOS	GGAATCTTGGAGCGAGTTGT	CCTCTTGTCTTTGACCCAGTAG	99
CD206	TGCAAAGCTATAGGTGGAGAGC	ACGGGAGAACCATCACTCC	164
IL-6	AGTTGCCTTCTTGGGACTGA	TCCACGATTTCCCAGAGAAC	159
CD115	TCATTCAGAGCCAGCTGCCCAT	ACAGGCTCCCAAGAGGTTGACT	560
STAT3	CAAGCCTTTCCTGACAGAGG	AGACAATGTCCTCACTGCCC	221
STAT6	CATCTGAACCGACCAGGAACT	CTCTGTGGGGCCTAATTTCCA	135
IL-6R	TGGGACCCGAGTTACTACTT	TGGATGACGCATTGGTACTG	110
β-Actin	AGAGGGAAATCGTGCGTGAC	CAATAGTGATGACCTGGCCGT	138

### Immunoblotting

Cells were lysed in RIPA lysis buffer (50 mmol/l Tris-HCl, pH 7.4, 150 mmol/l NaCl, 10 mmol/l phenylmethylsulfonylfluoride, 1 mmol/l EDTA, 0.1% SDS, 1% Triton X-100, 1% sodium deoxycholate) for 30–40 min on ice. Protein concentrations were determined using Pierce™ BCA protein assay kit (Thermo Scientific). Proteins were resolved by SDS-PAGE and then transferred to polyvinylidene fluoride membranes (Millipore, VIC, Australia). Membranes were blocked for 1 h at room temperature in 5% skim milk in 0.1% TBS/0.1% Tween20 and then incubated overnight with rabbit polyclonal antibodies to IL-6Rα (Sino Biological, Beijing, China), gp130 (R&D Systems), phospho-STAT6 (R&D Systems) and phospho-STAT3 (Cell Signaling Technology), STAT-3 (Cell Signaling Technology, QLD, Australia), and goat polyclonal antibodies to STAT6 (R&D systems). β-Actin (Abcam) was used as loading control.

Membranes were incubated with appropriate antigoat or antirabbit secondary antibodies (Santa Cruz, QLD, Australia) for 1 h at room temperature. Membranes were washed, incubated with Western Lightning Plus Enhanced Chemiluminescence Solution (PerkinElmer, Woodbridge, ON, Canada) for 1 min and exposed to Amersham Imager 600 (GE Healthcare Life Sciences, NSW, Australia) for 5 s to 10 min. For the analysis of the ratio of phosphorylated/non-phosphorylated transcription factors blots were stripped using mild conditions (Thermo Fisher Scientific) and reprobed with the appropriate antibodies. The density of the specific bands was quantified using Image J software (ImageJ, USA).

### Statistical Analysis

Statistical evaluation of liver weight, limiting dilution, RT-PCR, and immunohistochemical analysis results were presented as mean ± SD, as appropriate. Results were analyzed using GraphPad prism 5 software (Graphpad Software, San Diego, CA, USA) by Mann–Whitney tests for samples with unknown and potentially disparate variances, or by one-way ANOVA followed by *post hoc* analysis with Tukey’s test with *p* < 0.05 accepted as a level of statistical significance.

## Discussion

Tumor necrosis factor is a pleiotropic cytokine originally named after its proposed tumoricidal effects that has since been identified as a central effector cytokine with a broad range of biological activities such as induction of cell death, modification of cell migration, and regulation of DCs differentiation *in vitro* (24). Interestingly, its presence has been shown to be irreplaceable for effective immune responses to the bacterial or parasitic intracellular pathogens such as *Mycobacterium tuberculosis, Listeria monocytogenes*, or *L. major* but the underlying mechanisms that lead to this susceptibility are still not clear (10, 25–27).

After deletion of the *tnf* gene, normally resistant B6.WT mice are unable to control a cutaneous infection with *L. major* BNI (10). They develop a progressive infection that ultimately spreads to visceral organs including the liver. In our detailed analysis of this organ during *L. major* infection, we found that after day 21 after infection the size in B6.TNF^−/−^ mice was enlarged significantly and increasing numbers of viable *L. major* parasites could be detected while B6.WT controls essentially remained parasite free. The progressive infection that was caused by the absence of TNF was accompanied by a strong increase in the proportion of Mo-Ms that showed clear signs of alternative activation. The marked expression of IL-6 prompted us to analyze the interaction of IL-6 and TNF during macrophage differentiation and the signaling pathways *via* IL-6Ra, gp130, and STAT3 and 6 *in vitro*. Our results point to a role of IL-6 facilitating macrophage differentiation downstream of TNF.

Macrophages, while acting as a major reservoir for *L. major* parasites (28), are also potent effector cells that kill parasites *in vivo* with a strong production of NO (29, 30). In the skin and draining local lymph nodes, the tissues predominantly analyzed in *L. major* infection, resident macrophages are initially relevant for the immune response but are quickly outnumbered by inflammatory monocyte derived DCs (31–33). In the liver, which constitutes a major target organ of a visceralized *L. major* infection in immune-incompetent mice, the inflammatory infiltration of monocytic cells has not yet been addressed in detail. In our study, we defined tissue resident KC, recruited inflammatory monocytes (Mo) and the cell types Mo differentiate into, Mo-DCs and Mo-M according to their expression of the marker molecules CD45, F4/80, CD11b, and Ly6C (13–15) and analyzed the dynamic of these cell populations in response to *L. major* infection in the absence of TNF. Fate-mapping has demonstrated that liver-resident F4/80^+^ KC are derived from embryonic cells and self-maintain independently from hematopoietic input under non-inflammatory steady state conditions (34). These cells have been shown to have an anti-inflammatory function that is abrogated by inflammation (35). Only hours after inoculation with *L. monocytogenes*, KC become infected and rapidly undergo necroptosis, thereby triggering the recruitment of monocytes (13). Unsurprisingly, in the slower moving *L. major* infection we did not see a significant decline of the numbers of KC nor did we detect a significant genotype-dependent changes between B6.WT and B6.TNF^−/−^ during the course of infection (with the exception of day 21 after infection). Interestingly, while the number of liver Mo increased in correlation with the lesion size but independent of the mouse genotype, the population of Mo-M saw a significant increase in TNF-deficient mice but was hardly detectable in B6.WT mice. Since Mo-M in the liver differentiate out of the population of Mo in response to inflammatory signals (13) this observation could point to an absence of these signals in B6.WT mice due to an infection that is contained in skin and draining LN or potentially, to a contributing role of TNF in the differentiation (12).

Monocytes are present in form of steady-state precursors in the peripheral circulation and are recruited to organs such as the liver during inflammation (34). Here they differentiate to Mo-M and Mo-DCs in response to inflammatory cues. In the experiments presented here, the population of Mo increased after day 35 p.i. in line with the increased footpad swelling in both groups of mice. Numbers of Mo began to decrease at day 42 p.i. in B6.WT indicating that during the resolution phase of infection the recruitment of Mo ceased. However, in B6.TNF^−/−^ mice Mo continued to be recruited and to differentiate into Mo-M resulting in a strong presence of this cell type in the late phase of infection. Like TNF-deficient mice the highly susceptible BALB/c mice fail to control leishmaniaisis. However, Mo-M are not found in *L major* infected BALB/c mice, which supports the notion that an accumulation of Mo-M is due specifically to the absence TNF. In previous experiments, a similar Mo-M population was observed in the skin and the draining lymph node of B6.TNF^−/−^ mice during *L. major* BNI infection. It exhibited a phenotype that was CD11b^+^Ly-6C^low^CCR2^low^iNOS^low^ and harbored a large number of parasites (11). In further detailed mechanistic analyzes it could be demonstrated that these cells coexpressed iNOS with high levels of CD206 and Arg-1, which indicated an M2-like phenotype (12). This finding was seemingly contradicting a long line of publications that showed that TNF was supporting the expression of iNOS. The role of TNF in the induction of iNOS and the effector molecule NO during the innate immune response to *L. major* was investigated initially in *in vitro* models (36). It could be shown that it activated macrophages and synergized with IFN-γ to induce effector functions (8, 37). However, in more complex *in vivo* infection models, the well-defined role of TNF in the iNOS-inducing cytokine network determined *in vitro* became controversial (38) while the central effector role of NO remained undisputed (29, 30). A *L. major* infection of a TNFR1-negative mouse strain showed that in absence of this proinflammatory signaling pathway these mice developed persistent lesions but controlled the pathogen (38). Further investigations, using TNFR2- and TNFR1/2-deficient mice clearly demonstrated that the TNFR2 signaling pathway lacked a clear inflammatory function while the outcome of the infection of mice deficient for both TNFR1 and TNFR2 was similar to the TNFR1-negative strain (39). Unexpectedly, it became clear that the expression of iNOS was sustained in these mice (39). Finally, infection experiments using genetically pure TNF-negative C57BL/6 mice resulted in a progressive and ultimately fatal, infection (10) despite a strong Th1 response which was characterized by a hyper-expression of IFN-γ and the presence of iNOS (9, 11). The differences in the published clinical outcomes of these infection experiments were probably due to variations of the genetic background of the parasite strains (40) in combination with a contaminating presence of congenic regions (41) in the genomes of the TNFR1- and 2-deficient mice (39).

Recently, the apparent contradiction of the presence of iNOS in TNF-deficient mice and their concurrent susceptibility to *L. major* BNI infection could be explained with the observation that TNF caused a direct suppression of Arg-1 expression and of other molecules associated with an alternative activation of myeloid cells. In TNF-negative mice the number of Arg1^+^ cells was increased in skin and draining LN and in the absence of TNF a coexpression of Arg-1 and iNOS could be detected. Since both enzymes share l-arginine as substrate a coexpression in macrophages caused competition for the substrate. Consequently, loss of TNF significantly reduced the production of NO, resulting in fatal leishmaniasis (12). Interestingly, in this model the CD11b^+^ Arg1^+^ cells isolated from skin and draining LN of *L. major* BNI-infected B6.WT and B6.TNF^−/−^ mice were predominately coexpressing CD11c^+^ (12) and had therefore a phenotype that had been described earlier in the leishmanial model (42). This aspect was not addressed in the present study but it should be noted that the concept of inflammatory versus alternative activation can also be observed in DC (43). Taken together, our conclusive detection of M2 macrophages in the liver has strengthened the concept that the M2-suppressing role of TNF is not organ- or tissue-specific and, together with the observation of M2-like cells in the spleen of *L. monocytogenes* infected TNF-deficient mice, supports the notion of a fundamental, yet so far undescribed biological activity of this cytokine (12, 44, 45).

In the present study, we confirmed that IFN-γ was detectable at a higher level in B6.TNF^−/−^ mice than in B6.WT mice (9). Similarly, MCP-1 (46), a pivotal cytokine implicated in recruitment and activation of monocytes (47) was overexpressed in the serum of TNF-deficient mice after *L. major* BNI infection. However, most interestingly, we could show that the major proinflammatory cytokine IL-6 was significantly overexpressed. Historically, after it had been demonstrated that these cytokines were involved in the acute phase reaction (48) this pleiotropic cytokine has been grouped with IL-1 and TNF as a classical proinflammatory mediator that appears early in the immune response (49). The interplay of TNF and IL-6 has been addressed in *in vitro* experiments using DC and macrophages. An activation of TNF-negative bone marrow derived DC resulted in the secretion of a decreased amount of IL-10 but IL-6 production remained unchanged (50). An infection with *L. major* or *L. donovani* with subsequent activation with LPS resulted in a strong IL-6 expression (51). Finally, IL-6 can downregulate the expression of proinflammatory cytokines including TNF (52) and it has been described to skew the differentiation of monocytes to macrophages (53). This action can be reversed by the additional presence of TNF (16) and this activity is caused by opposite effects of these cytokines on M-CSF receptor expression and internalization. One major activities of IL-6 could potentially be important *in vivo* during the immune response to *L. major* BNI. The presence of IL-6 has been reported to block the differentiation of regulatory T cells (Tregs) and to support the generation of IL-17^+^ Th cells (Th17) (54). However, an infection of mice which were genetically deficient for IL-6 with *L. major* demonstrated an effective and protective antileishmanial response (55) despite originally having been described to have an impaired antibacterial, antiviral, and acute phase response (56); a more detailed analysis of the adaptive and the innate branches of the immune system could not detect major deficiencies in these mice (57).

In the classical IL-6 signaling pathway, IL-6 signal transduction requires the formation of a trimer consisting of ligand, the IL-6 receptor (IL-6R) α-chain and the signal transducing membrane glycoprotein gp130. IL-6 binds to membrane-bound IL-6R (mIL-6Rα) which recruits its signaling component gp130. This combination contributes to the plethora of pro- and anti-inflammatory functions (58). Unlike the ubiquitously distributed gp130, mIL-6Rα is expressed exclusively on the surface of hepatocytes and some myeloid and lymphoid cell populations such as monocytes and T cells (59). However, most proinflammatory functions of IL-6 are mediated through a second IL-6-dependent signaling pathway *via* the soluble IL-6R in the IL-6 trans-signaling pathway which can trigger IL-6-mediated response in cells negative for mIL-6R (58).

In our study, stimulation with IL-6 increased the expression of mIL-6R and gp130 but the additional presence of TNF caused a rapid downregulation of these receptor molecules and a significant reduction of STAT3 and 6 phosphorylation. In the context a *L. major* infection of TNF-competent animals, the role of IL-6 in the determination of the outcome of macrophage differentiation seems to be negligible (57). This changes however, in the absence of TNF. Now a strong expression of IL-6 contributes significantly to the dysregulation that leads to a fatal presence of M2 macrophages in skin (12), lymphoid organs and visceral organs such as the liver. Potentially our finding could have ramifications for the meanwhile ubiquitous anti-TNF and IL-6 therapy in human chronic inflammatory disorders. Therefore, the complex regulatory interactions of IL-4, IL-6, and TNF in macrophage differentiation need to be investigated at the molecular level in more detail (12, 16, 53).

Taken together, we show that in the absence of TNF an infection with *L. major* BNI quickly spreads to the liver. The infection causes a strong infiltration of monocytes and leads to the presence of an accumulating number of Mo-Ms which display an M2 phenotype, express IL-6 and harbor parasites which contributes to the fatal outcome of leishmaniasis in these mice. Furthermore, we show that in the absence of the M1 determining cytokine TNF, anti-inflammatory function of IL-6 can dominate the differentiation of monocytes and provide a first insight that this cytokine can contribute to the skewing of monocytes to a M2 phenotype.

## Ethics Statement

Animal care and experiments were approved by the animal ethics committee of the University of Tasmania, Hobart, Australia (Animal Ethics Numbers: A13934 and A13935).

## Author Contributions

SH: designed experiments, carried out experiments, acquired data, analyzed data, and edited the manuscript. CM and JD: carried out experiments, analyzed data, and edited the manuscript. WW: edited the manuscript and revised the final version. AL: designed experiments, edited the manuscript, and revised the final version. HK: conception of the project, designed experiments, analyzed data, wrote the manuscript, edited the manuscript, and revised the final version.

## Conflict of Interest Statement

The authors declare that the research was conducted in the absence of any commercial or financial relationships that could be construed as a potential conflict of interest.

## Supplementary Material

Figure S1 Macrophages derive from recruited monocytes. Flow cytometric gating strategy and analysis of the three distinct subsets of CD45^+^ F4/80^+^ liver macrophages after *L. major* BNI infection.

Figure S2 Analysis by immunofluorescence and flow cytometry in *L. major*-infected BALB/c mice. **(A)** Immunofluorescence staining of CD11b (gray), iNOS (green), *L. major* (red), and DAPI (blue) in liver tissue of BALB/c mice postinfection. Results represent one of three biological repeats. **(B)** Flow cytometric analysis revealed the changes of three different CD45^+^F4/80^+^ liver macrophage populations based on identical gates as in Figure 3 over the course of *L. major* BNI infection.

Figure S3 The expression of CD11c on monocytic cells from B6.WT and B6.TNF^−/−^ mice. **(A)** Flow cytometric analysis of liver Ly6C^+^CD11b^+^ cells obtained from *L. major* BNI*-*infected B6.WT and B6.TNF^−/−^ mice. The populations of Ly6C^+^CD11b^+^ cells were gated and the CD11c expression of these cells is shown in a representative example. **(B)** Quantification by flow cytometry of the expression of CD11c on the Ly6C^+^CD11b^+^ cells upon *L. major* BNI infection. The data represent the median of mean intensity of fluorescence (MIF) of CD11c expression by Ly6C^+^CD11b^+^ cells upon *L. major* BNI infection. Results represent means ± SD, *n* = 15. **p* < 0.05 comparing to B6.WT group, two-tailed Mann–Whitney *U*-test.

Figure S4 The morphology of Mo and Mo-M from B6.WT and B6.TNF^−/−^ mice. **(A)** Representative Romanowsky (Diff-Quik) stain of liver sections are shown (*n* = 3 per group). After day 42 p.i., *L. major* BNI parasites were found inside and outside of the cells (arrowheads) in the liver of B6.WT and B6.TNF^−/−^ mice (magnification 400×).
