# Supplementary figures and images for: Absence of Tumor Necrosis Factor Supports Alternative Activation of Macrophages in the Liver after Infection with Leishmania major
Source: Front Immunol. 2018 Jan 19;9:1. doi: 10.3389/fimmu.2018.00001 (PMC5780437; doi:10.3389/fimmu.2018.00001)

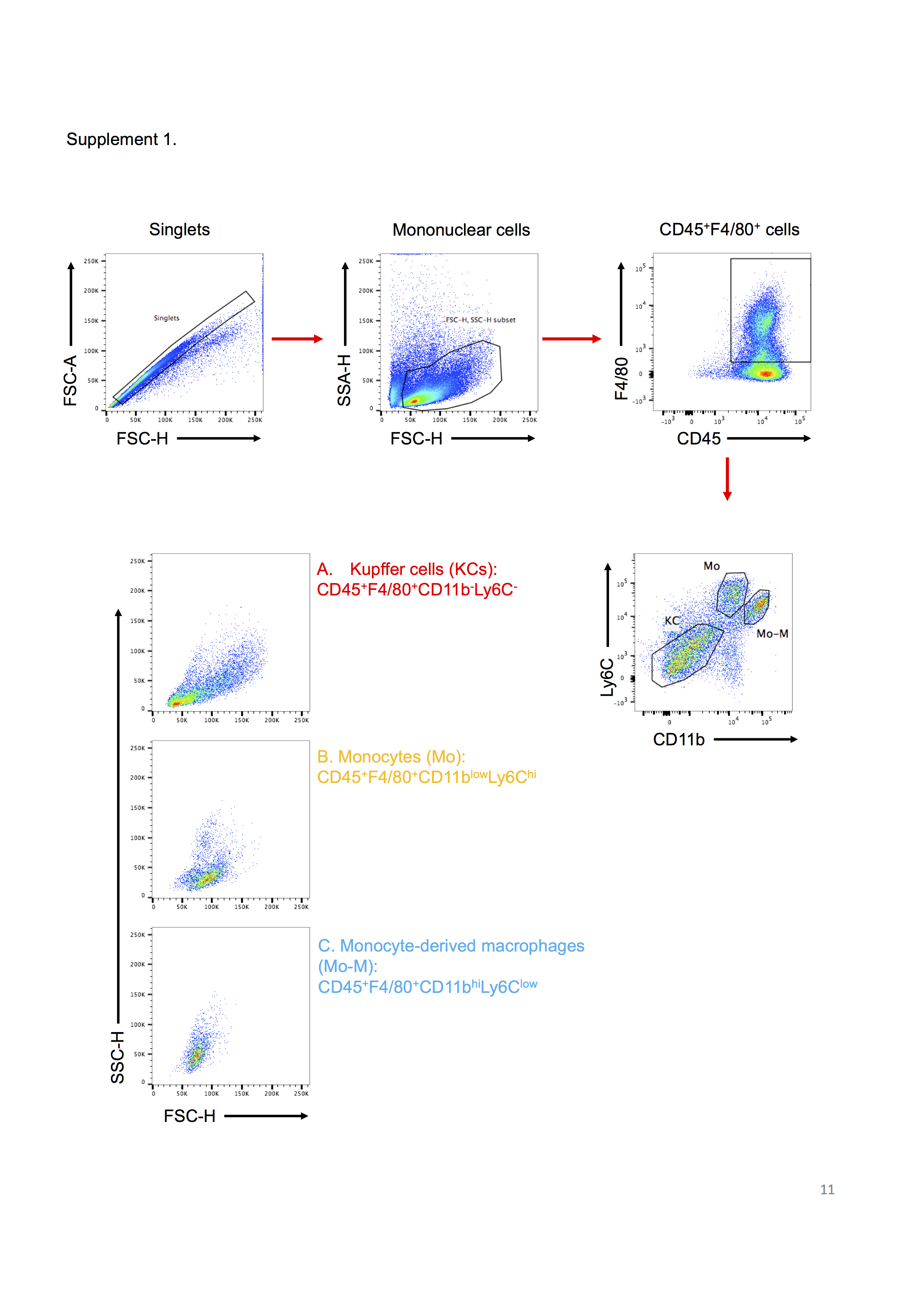

Supplement: Figure S1 — Macrophages derive from recruited monocytes. Flow cytometric gating strategy and analysis of the three distinct subsets of CD45+ F4/80+ liver macrophages after L. major BNI infection. [file Image_1.jpg]

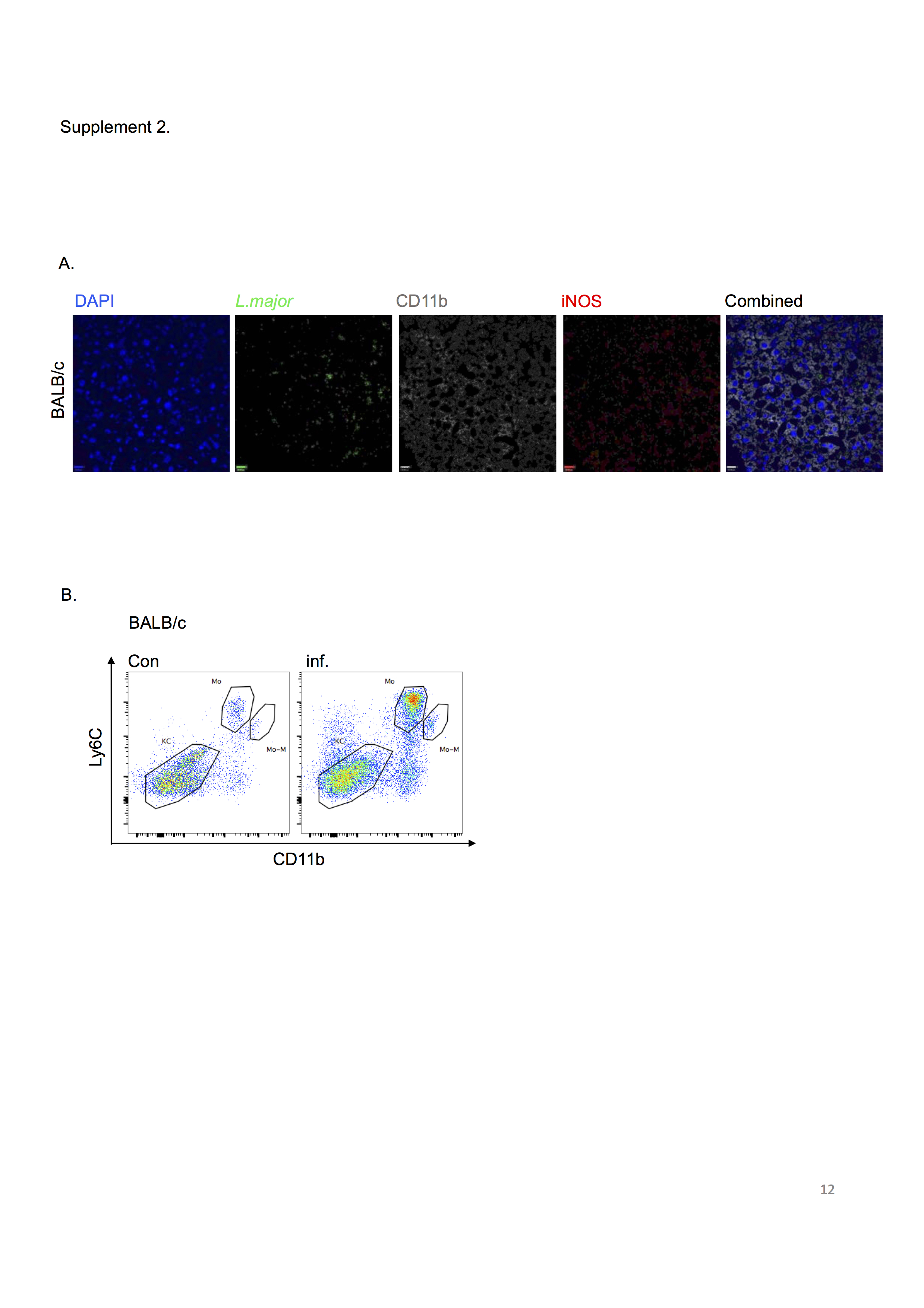

Supplement: Figure S2 — Analysis by immunofluorescence and flow cytometry in L. major-infected BALB/c mice. (A) Immunofluorescence staining of CD11b (gray), iNOS (green), L. major (red), and DAPI (blue) in liver tissue of BALB/c mice postinfection. Results represent one of three biological repeats. (B) Flow cytometric analysis revealed the changes of three different CD45+F4/80+ liver macrophage populations based on identical gates as in Figure 3 over the course of L. major BNI infection. [file Image_2.jpg]

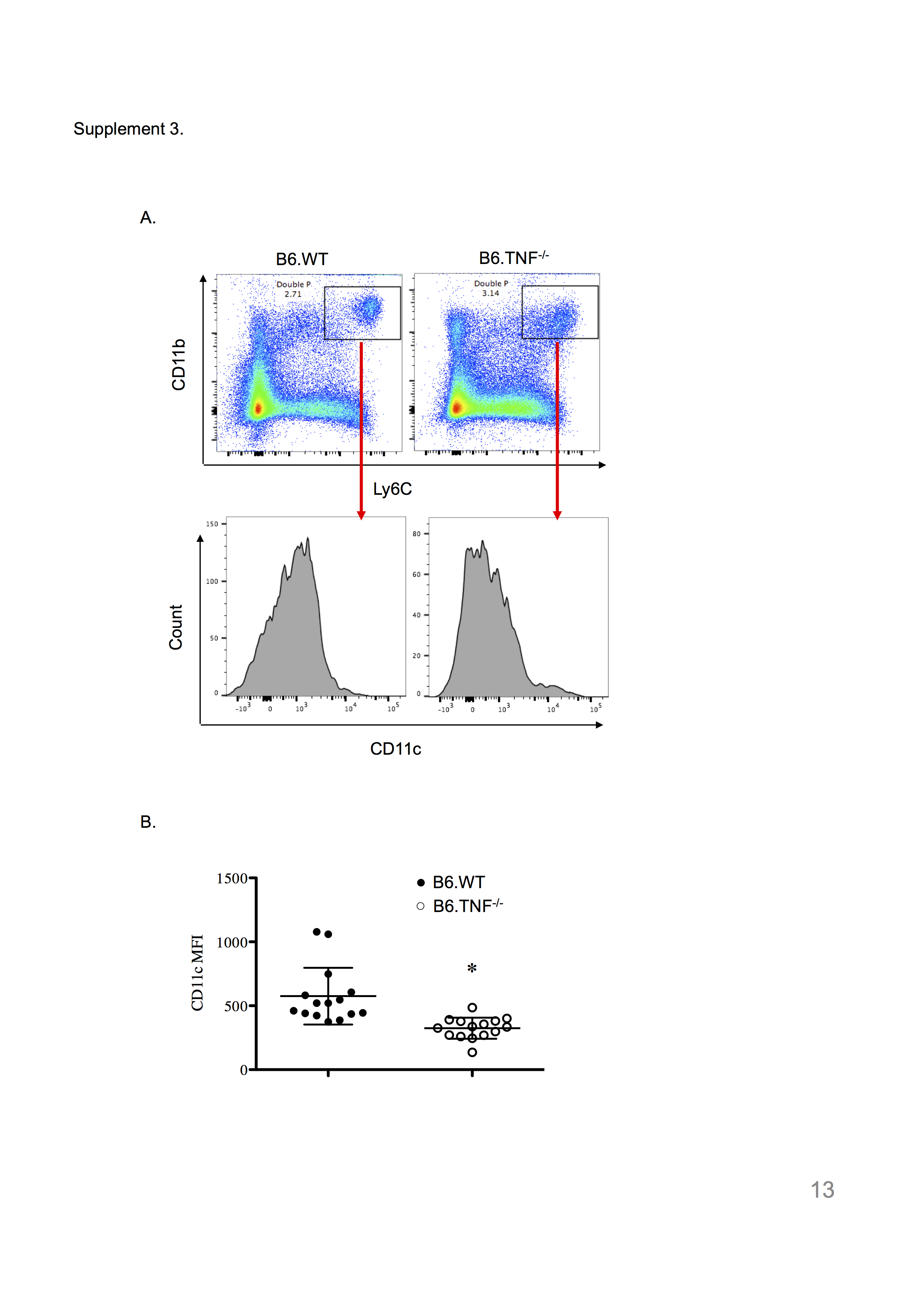

Supplement: Figure S3 — The expression of CD11c on monocytic cells from B6.WT and B6.TNF−/− mice. (A) Flow cytometric analysis of liver Ly6C+CD11b+ cells obtained from L. major BNI-infected B6.WT and B6.TNF−/− mice. The populations of Ly6C+CD11b+ cells were gated and the CD11c expression of these cells is shown in a representative example. (B) Quantification by flow cytometry of the expression of CD11c on the Ly6C+CD11b+ cells upon L. major BNI infection. The data represent the median of mean intensity of fluorescence (MIF) of CD11c expression by Ly6C+CD11b+ cells upon L. major BNI infection. Results represent means ± SD, n = 15. *p < 0.05 comparing to B6.WT group, two-tailed Mann–Whitney U-test. [file Image_3.jpg]

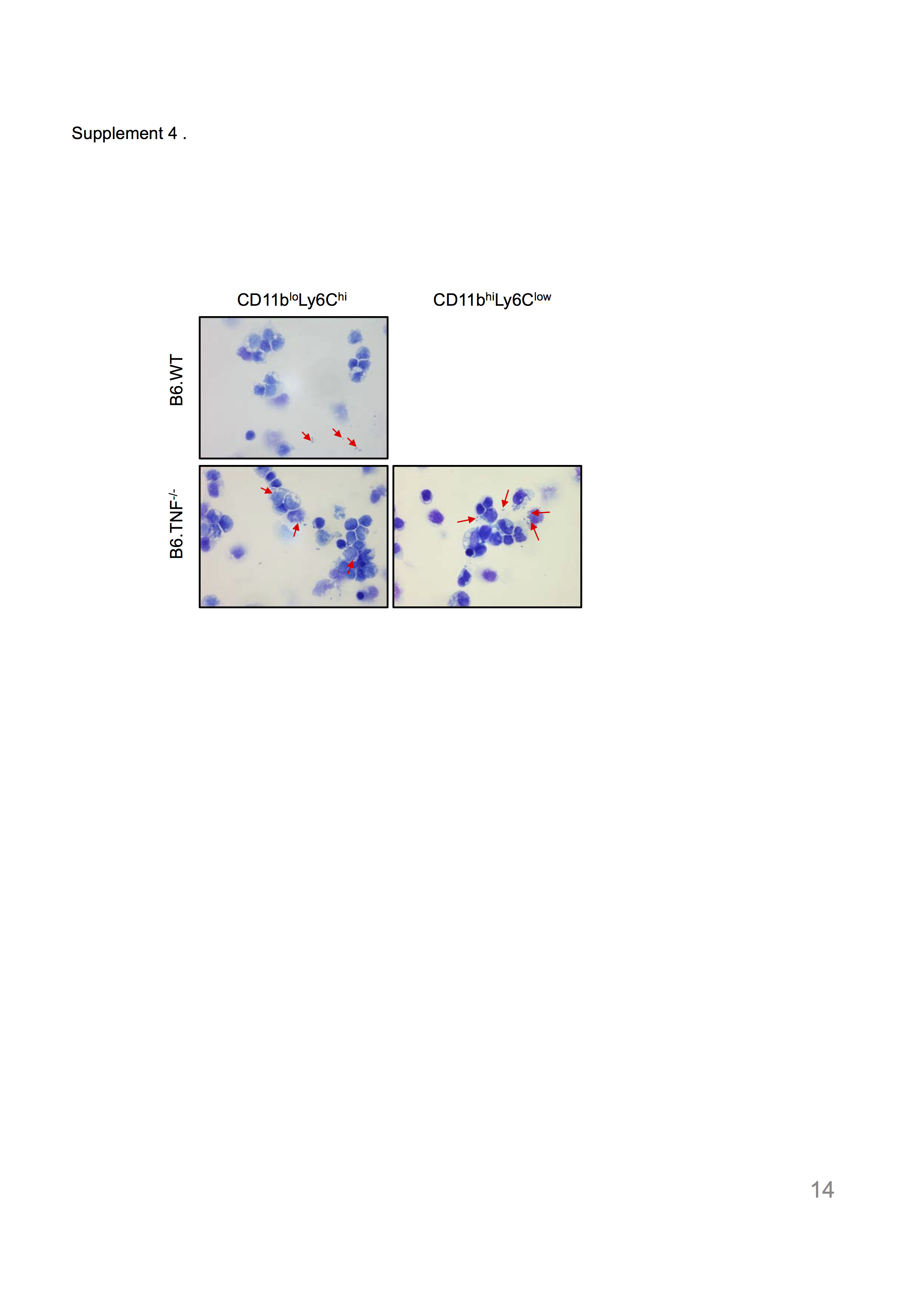

Supplement: Figure S4 — The morphology of Mo and Mo-M from B6.WT and B6.TNF−/− mice. (A) Representative Romanowsky (Diff-Quik) stain of liver sections are shown (n = 3 per group). After day 42 p.i., L. major BNI parasites were found inside and outside of the cells (arrowheads) in the liver of B6.WT and B6.TNF−/− mice (magnification 400×). [file Image_4.jpg]
